# Direct provision versus facility collection of HIV self-tests among female sex workers in Uganda: A cluster-randomized controlled health systems trial

**DOI:** 10.1371/journal.pmed.1002458

**Published:** 2017-11-28

**Authors:** Katrina Ortblad, Daniel Kibuuka Musoke, Thomson Ngabirano, Aidah Nakitende, Jonathan Magoola, Prossy Kayiira, Geoffrey Taasi, Leah G. Barresi, Jessica E. Haberer, Margaret A. McConnell, Catherine E. Oldenburg, Till Bärnighausen

**Affiliations:** 1 Department of Global Health and Population, Harvard T.H. Chan School of Public Health, Boston, Massachusetts, United States of America; 2 International Research Consortium, Kampala, Uganda; 3 Uganda Health Marketing Group, Kampala, Uganda; 4 Ugandan Ministry of Health, Kampala, Uganda; 5 Department of Epidemiology, Harvard T.H. Chan School of Public Health, Boston, Massachusetts, United States of America; 6 Department of General Internal Medicine, Massachusetts General Hospital, Boston, Massachusetts, United States of America; 7 Francis I. Proctor Foundation, University of California, San Francisco, San Francisco, California, United States of America; 8 Department of Ophthalmology, University of California, San Francisco, San Francisco, California, United States of America; 9 Department of Epidemiology & Biostatistics, University of California, San Francisco, San Francisco, California, United States of America; 10 Africa Health Research Institute, KwaZulu-Natal, South Africa; 11 Heidelberg Institute of Public Health, University of Heidelberg, Heidelberg, Germany; University of California, San Francisco, UNITED STATES

## Abstract

**Background:**

HIV self-testing allows HIV testing at any place and time and without health workers. HIV self-testing may thus be particularly useful for female sex workers (FSWs), who should test frequently but face stigma and financial and time barriers when accessing healthcare facilities.

**Methods and findings:**

We conducted a cluster-randomized controlled health systems trial among FSWs in Kampala, Uganda, to measure the effect of 2 HIV self-testing delivery models on HIV testing and linkage to care outcomes. FSW peer educator groups (1 peer educator and 8 participants) were randomized to either (1) direct provision of HIV self-tests, (2) provision of coupons for free collection of HIV self-tests in a healthcare facility, or (3) standard of care HIV testing. We randomized 960 participants in 120 peer educator groups from October 18, 2016, to November 16, 2016. Participants’ median age was 28 years (IQR 24–32). Our prespecified primary outcomes were self-report of any HIV testing at 1 month and at 4 months; our prespecified secondary outcomes were self-report of HIV self-test use, seeking HIV-related medical care and ART initiation. In addition, we analyzed 2 secondary outcomes that were not prespecified: self-report of repeat HIV testing—to understand the intervention effects on frequent testing—and self-reported facility-based testing—to quantify substitution effects. Participants in the direct provision arm were significantly more likely to have tested for HIV than those in the standard of care arm, both at 1 month (risk ratio [RR] 1.33, 95% CI 1.17–1.51, *p* < 0.001) and at 4 months (RR 1.14, 95% CI 1.07–1.22, *p* < 0.001). Participants in the direct provision arm were also significantly more likely to have tested for HIV than those in the facility collection arm, both at 1 month (RR 1.18, 95% CI 1.07–1.31, *p* = 0.001) and at 4 months (RR 1.03, 95% CI 1.01–1.05, *p* = 0.02). At 1 month, fewer participants in the intervention arms had sought medical care for HIV than in the standard of care arm, but these differences were not significant and were reduced in magnitude at 4 months. There were no statistically significant differences in ART initiation across study arms. At 4 months, participants in the direct provision arm were significantly more likely to have tested twice for HIV than those in the standard of care arm (RR 1.51, 95% CI 1.29–1.77, *p* < 0.001) and those in the facility collection arm (RR 1.22, 95% CI 1.08–1.37, *p* = 0.001). Participants in the HIV self-testing arms almost completely replaced facility-based testing with self-testing. Two adverse events related to HIV self-testing were reported: interpersonal violence and mental distress. Study limitations included self-reported outcomes and limited generalizability beyond FSWs in similar settings.

**Conclusions:**

In this study, HIV self-testing appeared to be safe and increased recent and repeat HIV testing among FSWs. We found that direct provision of HIV self-tests was significantly more effective in increasing HIV testing among FSWs than passively offering HIV self-tests for collection in healthcare facilities. HIV self-testing could play an important role in supporting HIV interventions that require frequent HIV testing, such as HIV treatment as prevention, behavior change for transmission reduction, and pre-exposure prophylaxis.

**Trial registration:**

ClinicalTrials.gov NCT02846402

## Abstract 2

In a cluster-randomized trial, Katrina Ortblad and colleagues study the provision of HIV self-tests for female sex workers in Uganda.

## Author summary

**Why was this study done?:**

- HIV self-testing can increase the convenience and privacy of HIV testing because it allows individuals to test for HIV at any time and place they choose and without the presence of another person.
- Many sub-Saharan governments are considering HIV self-testing for FSWs, but little evidence exists on the delivery, uptake, and effects of HIV self-testing among members of this population.

**What did the researchers do and find?:**

- We randomized 960 FSWs in 120 peer educator groups 1:1:1 to (1) direct provision of HIV self-tests, (2) provision of a coupon for free collection of HIV self-tests in a healthcare facility, and (3) standard of care facility-based HIV testing services. Participants received 1 HIV self-test (in the direct provision arm) or 1 coupon (in the facility collection arm) shortly after randomization and again 3 months later.
- Any recent HIV testing (at 1 month and at 4 months) and repeat HIV testing (at 4 months) was significantly greater in the direct provision arm compared to the facility collection arm and the standard of care arm.
- FSWs in the intervention arms almost completely substituted HIV self-testing for facility-based HIV testing.
- Fewer FSWs in the interventions arms sought HIV-related medical care than those in the standard of care arm at 1 month; however, this difference disappeared by 4 months.

**What do these findings mean?:**

- HIV self-testing appears to be safe and increases recent and repeat HIV testing among FSW in Uganda.
- The active approach to directly provide HIV self-tests to FSWs is more effective in increasing HIV testing than the passive approach to offer free access to HIV self-tests via coupons that can be used to collect self-tests in healthcare facilities.
- Almost all FSWs who had access to HIV self-testing used this new testing technology instead of facility-based HIV testing.
- It is possible that self-testing in homes and other private locations (rather than in healthcare facilities) may reduce linkage to care in the short term.
- Sub-Saharan African governments should consider HIV self-testing for FSWs but ensure it is delivered with linkage-enhancing interventions. Where possible, governments should introduce active HIV self-testing models (delivering HIV self-tests directly to FSWs) rather than passive models (requiring FSWs to collect self-tests in healthcare facilities).

## Introduction

HIV testing is the important first step for both HIV treatment and HIV prevention interventions [1–5]. In sub-Saharan Africa, 20%–30% of people who are HIV positive still do not know their HIV status and can thus not utilize lifesaving HIV treatment [1,2,5–9]. Among people who are HIV-negative, frequent HIV testing is key to HIV prevention. In particular, frequent HIV testing is a necessary condition for treatment as prevention (TasP) [3,10], behavior change to prevent transmission [11,12], and HIV pre-exposure prophylaxis (PrEP) [4,13].

Frequent HIV testing is especially important for those key populations that face the highest HIV risks. In sub-Saharan Africa, the largest key population is female sex workers (FSWs) and their clients [1]. FSWs in sub-Saharan Africa have an approximately 5 times higher prevalence of HIV than the general population [14–17]. The World Health Organization recommends that FSWs test for HIV every 3 months [18,19], but many FSW communities in sub-Saharan Africa do not achieve this standard [20–22]. Frequently cited barriers to HIV testing for FSWs include healthcare provider stigma and discrimination [23–26], transport costs [16], and inconvenient location [16,24] and opening hours of testing centers [24]. HIV self-testing may allow FSWs to overcome several of these barriers: it does not require an interaction with a health worker, it does not require travel to a facility, and it can be carried out in any space and at any time.

Despite these advantages, few sub-Saharan African countries have introduced HIV self-testing because of concerns related to the specific features of self-testing: it decouples testing from standard pre- and post-test HIV counselling and it places testing outside healthcare facilities, in which other services are available [27,28]. As a result, HIV self-testing may lead to more frequent social or emotional harms following a test result and decrease linkage to HIV treatment and prevention services. Another important reason for governments’ reluctance to commit to HIV self-testing policies is that evidence on the impact of HIV self-testing on key populations is currently lacking [27,28].

The uptake of HIV self-testing will likely depend on the approach that is used to deliver it. The delivery model will determine the extent to which HIV self-testing can overcome the different types of barriers to HIV testing, such provider stigma, transport costs and inconvenient locations and opening hours of testing centers. In this study, we aimed to establish the effectiveness of HIV self-testing in increasing recent and repeat HIV testing among FSWs using 2 delivery models: (1) direct peer provision of an oral HIV self-test and (2) peer provision of a coupon for free collection of an oral HIV self-test in a healthcare facility.

We used FSWs who were trained as peer educators for this study because they have good access to other FSWs and are able to engage with FSWs who do not normally utilize the health system. FSWs tend to trust other FSWs, and trust is important in the context of introducing a new healthcare intervention and technology that may initially be perceived as threatening [29]. Additionally, FSW peer educators are a realistic platform for the future scale-up of HIV self-testing strategies [30]; they have previously been used to deliver many types of health interventions to FSWs in sub-Saharan African [30–33].

The direct provision of HIV self-tests to FSWs fully realizes the advantages of self-testing (i.e., testing that is independent of facility hours and location and does not require a health worker or any other person). A disadvantage of this model is, however, that it decouples HIV testing from the health system, in which counseling and HIV treatment and prevention services are provided. Without counselling following an HIV test, mental distress may be more common; without proximity to HIV treatment services, linkage to care may be delayed.

Facility-based collection of HIV self-tests is more like standard facility-based testing than the direct provision of HIV self-tests because it requires FSWs to go to a healthcare facility to be able to test for HIV. We included facility-based collection in our study because it does allow FSWs to freely choose the time and place of testing and because it closely resembles the standard HIV self-testing model that many countries will adopt—passive provision of HIV self-tests in facilities rather than active delivery of HIV self-tests to FSWs by peer educators.

A number of exploratory studies in sub-Saharan Africa have shown high acceptability and good performance of HIV self-testing [34–44]. Several HIV self-testing trials have been carried out in the general population [45] and in 2 key populations—the male partners of women attending antenatal care [46–49] and men who have sex with men [49–53]. Jointly with a concurrent study [54], this trial is to our knowledge the first to investigate the effects of HIV self-testing among FSWs in sub-Saharan Africa.

## Methods

### Ethics statement

Ethical approval for the study was granted by the Institutional Review Board at the Harvard T.H. Chan School of Public Health (IRB16-0885) and Mildmay Uganda Research Ethics Committee (REF 0105–2016).

### Study setting

This study was conducted in Kampala, the capital city of Uganda, which has an estimated 13,000 FSWs [55,56]. About 1 in 3 FSWs in Uganda is HIV positive [55,56]. FSWs are a particular focus of the Ugandan Ministry of Health (MOH) for health and HIV interventions. In Kampala, FSWs have access to a range of HIV testing options through the country’s Most at Risk Populations Initiative (MARPI), including facility-, home-, and work-based HIV testing [57]. There are also 4 nongovernmental organizations (NGOs) operating within Kampala that use peer interventions to mobilize FSWs for HIV prevention and help provide health services and economic opportunities. To date, the Ugandan government has not issued a guideline or policy for HIV self-testing [58,59].

### Study design

This 3-arm cluster-randomized controlled trial was designed to establish the effect of 2 different HIV self-testing delivery models for FSWs on HIV testing and linkage outcomes. All participating FSWs were organized into peer educator groups (1 peer educator and 8 FSWs). Peer educator groups were randomized into 1 of 3 study arms: (1) direct provision of an HIV self-test, (2) a coupon for free collection of an HIV self-test in a healthcare facility, and (3) standard of care HIV testing. Our study protocol and the prespecified primary and secondary outcomes of this trial can be found in the clinical trials registry and database run by the United States National Library of Medicine at the National Institutes of Health, ClinicalTrials.gov (NCT02846402). The trial’s Manual of Operations and Procedures is included in the Supporting Information (**S1 Text**).

### FSW peer educators

All FSW peer educators in this study were affiliated with existing FSW NGOs or MARPI clinics throughout Kampala. There was no educational or age requirement for FSW peer educators; trust and respect within the local FSW community (determined by FSW NGO leaders and MARPI clinic coordinators) were the most important prerequisites. To ensure wide representation of FSWs in Kampala, an even number of peers (24) were recruited from each of the 5 Kampala divisions. Prior to participant enrollment, all peer educators completed a 2-day training during which they learned how to instruct participants to use the oral HIV self-test, interpret the results, and encourage linkage to HIV treatment and prevention services. The peer educators recruited potential study participants and referred them to research assistants, who first conducted a phone screening followed by an in-person eligibility assessment and enrollment. Peer educators were encouraged to recruit FSWs they already knew to ensure trust. We considered trust between the people delivering the trial interventions and the participants to be particularly important, because HIV self-testing is a new technology and might be feared to be harmful. The peer educators received 90,000 UGX per visit. At market exchange rates, this amount of UGX is about US$25; after adjusting for purchasing power parity (PPP), this amount of UGX equals about US$79 [60].

### Study participants

Participants were eligible for trial enrollment if they (1) were 18 years or older; (2) reported the exchange of any vaginal, anal, or oral sex for money, goods, or other items of value; (3) said that they had never tested for HIV or self-reported negative HIV status without a recent HIV test (in the past 3 months); (4) had been working as FSWs in Kampala for at least 1 month prior to enrollment and planned on continuing to work as FSWs in Kampala for at least the next 4 months; and (5) had never used an oral HIV self-test. All eligible participants provided written informed consent; participants had to be of sound mind (i.e., not under the influence of drugs or alcohol) to be eligible for the informed consent process.

### Peer educator groups

We used peer educators to recruit participants for our study. Peer educators are a common and tested approach for providing interventions to FSWs [27], including in public sector health systems in sub-Saharan Africa [30–33].

In Kampala, we had access to established FSW peer educators, who we could recruit and train to deliver our intervention. The use of peer educator groups in this trial guarded against intervention spillover effects. FSWs often cluster in particular locations (e.g., guest houses or brothels). Peers are likely to recruit from one such location and within pre-existing social networks. The peer educator-based trial assignment thus makes it more likely that any information (or HIV self-test) that is shared is shared among FSWs belonging to the same arm of the trial. The peer educator groups met once during the first peer educator visit; all subsequent peer educator visits were individual visits.

### Randomization procedures

We randomized FSW peer educator groups 1:1:1 to 1 of 3 study arms. One of the authors, CEO, developed the randomization list using R Studio (Version 3.3.1, The R Foundation for Statistical Computing, Vienna, Austria) [61]. To ensure balance across study arms, we stratified the list by Kampala’s 5 divisions and evenly recruited peers from the divisions. To increase the probability that equal numbers of FSWs were allocated to all 3 study arms within each division, we used block randomization (using block sizes of 3, 6, and 9). Once the peer educator groups had been formed, the research assistants opened sealed randomization envelopes to assign the groups to one of the trial arms. Research assistants, peer educators, and participants were masked to study arm assignment prior to opening the envelopes.

### Interventions

**Fig 1:**
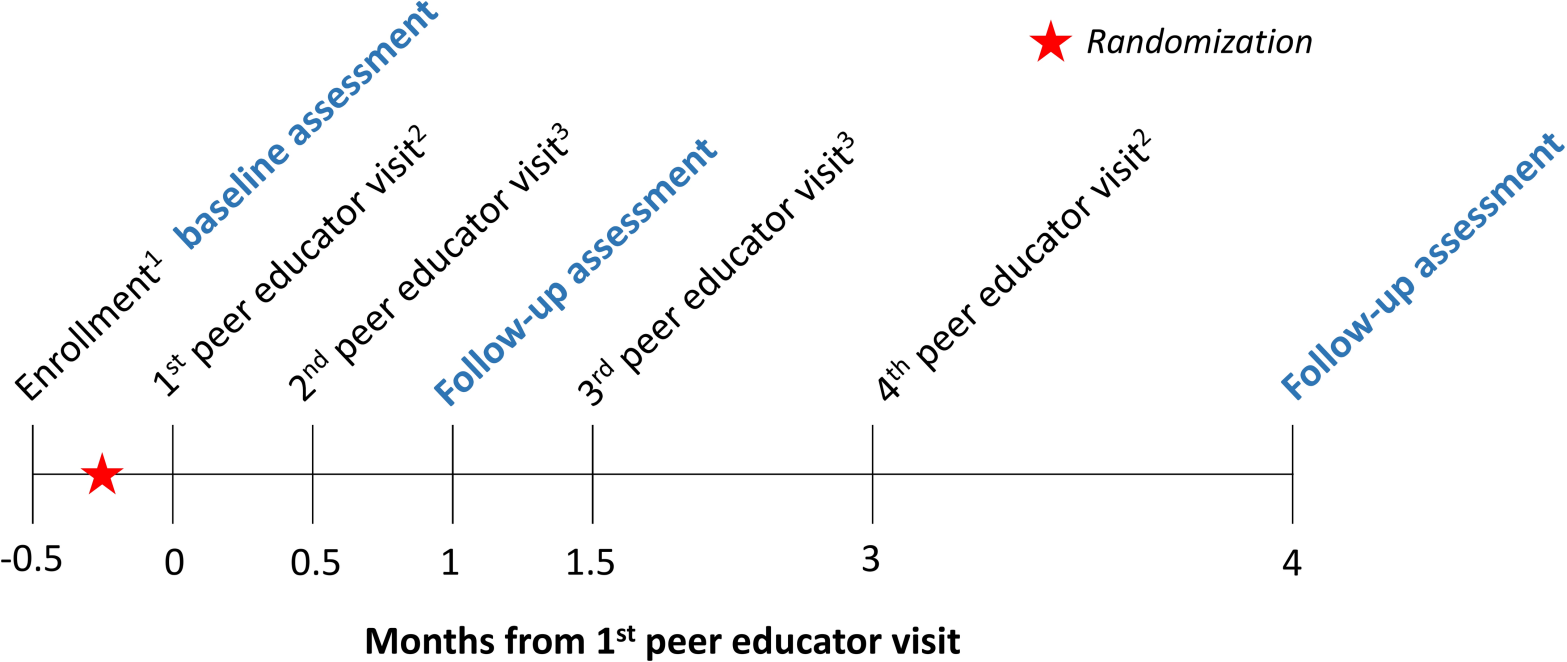
Time line of study interventions and assessments (conducted by research assistants, in blue). Participants were randomized in groups of 1 peer educator and 8 FSWs. The text following the subscripts below describe details about each peer educator visit: ^**1**^Research assistants gave all participants a referral card for free HIV testing and a study contact card. The referral card could be used at 10 private healthcare facilities participating in this study. The study contact card included a toll-free hotline number, which participants could call for information about linkage to care, to report potential adverse events, or to ask questions related to HIV self-testing (intervention arms only). ^2^The peer educators gave all participants condoms. In the direct provision arm, the peer educators additionally gave the participants oral HIV self-tests; in the facility collection arm, participants received coupons, which they could exchange for an HIV self-test at the participating healthcare facilities. ^3^The peer educators gave all participants condoms.

**Fig 1** shows the study intervention activities in chronological order. Research assistants enrolled eligible participants. The research assistants gave all participants a referral card for facility-based HIV testing and a study card. The referral card could be used for HIV testing in 10 participating private healthcare facilities, which were evenly dispersed throughout Kampala’s 5 divisions. The study card included a toll-free hotline number, which participants were encouraged to call for referral to standard of care HIV testing and treatment services and to report potential adverse events. Participants in the HIV self-testing intervention arms were also encouraged to call the hotline number if they had any questions or concerns related to HIV self-testing. Individuals working at the hotline received training on HIV self-testing and the study procedures prior to participant enrollment.

The peer educators were scheduled to visit participants 4 times over the duration of the study. At all visits, the peer educators were to distribute condoms, refer participants to standard of care HIV testing services (including all public and private HIV testing facilities), and screen for potential adverse events. The first peer educator visit was a group visit so that the peer educators in the HIV self-testing intervention arms could provide participants with HIV self-test training, including self-test use, results interpretation, and linkage to care. The peer educators were trained to instruct participants that they should go to a healthcare facility for confirmatory testing if they said that they were HIV positive. The peer educators were also trained to instruct participants to test again 3 months after testing HIV negative. Research assistants observed the first peer educator visits to ensure participants received consistent information on oral HIV self-testing. The following 3 peer educator visits (at 2 weeks, at 1.5 months, and at 3 months) were individual visits to safeguard the confidentiality of participants who had learned that they were HIV-positive or wanted to report adverse events (Fig 1). Research assistants were instructed to call peer educators before and after each scheduled visit to ensure the visits happened and happened on time.

The peer educators in the intervention arms were trained to give participants in the intervention arms either 2 oral fluid rapid HIV self-tests (direct provision arm) or 2 coupons for free HIV self-tests (facility collection arm) over the duration of the study. Participants were to receive the first HIV self-test or coupon at the first peer educator visit and the second 3 months later, at the fourth peer educator visit. We used the OraQuick Rapid HIV-1/2 Antibody Test (OraSure Technologies, Bethlehem, PA); each test included pictorial and written step-by-step instructions (in the local language, Luganda, as well as in English) on test use, results interpretation, and follow-up care.

The participants in the facility collection arm of this study could exchange the coupon they received for HIV self-tests in the 10 private healthcare facilities described above. All 10 healthcare facilities were affiliated with our implementing partner, the Ugandan Health Marketing Group, which enabled us to closely monitor the distribution of the oral HIV self-test. Prior to enrollment, the research assistants trained the health workers in these 10 facilities in oral HIV self-test use and the study procedures. To minimize the risk of stigma and discrimination, the healthcare workers also received FSW sensitization training, which included skill building on eye contact, appropriate body language, and nonjudgmental spoken language. Two of the 10 private healthcare facilities provided free antiretroviral treatment for people who were HIV-positive.

### Assessments

The research assistants conducted 3 quantitative outcome assessments with each study participant over the course of the study: a baseline assessment (post-enrollment and prior to randomization) and 2 follow-up assessments after the first peer educator visit (one at 1 month and the other at 4 months, **Fig 1**). The baseline assessment included questions on sociodemographic characteristics, sex work history, sexual behaviors, and past HIV testing (timing and location) as well as intimate partner violence (IPV). We measured both physical and sexual IPV. To measure physical IPV, we asked participants if a sexual partner had hit, slapped, punched, or shoved them or had done anything else to physically hurt them in the past month. To measure sexual IPV, we asked participants if they had sex against their will in the past month. The follow-up assessments included identical questions on sexual behavior, HIV testing, and IPV. Additionally, these follow-up assessments included questions about HIV self-test use and linkage to care. All data were collected electronically in face-to-face interviews using the CommCare electronic data platform (Dimagi Inc, Cambridge, MA). At the 4-month assessment, participants were offered free membership into a Kampala-based FSW organization (if they were not already a member). Upon completion of each assessment, participants received 16,500 Ugandan Shillings (UGX) (about 14 purchasing power parity [PPP]-adjusted US dollars [60]) as compensation for their time.

### Outcomes

Our prespecified primary outcomes were any HIV testing following the first peer educator visit, measured at 1 month and at 4 months. To measure these outcomes, we asked participants when they last tested for HIV (last month, 2–3 months, >3–6 months, >6–12 months, >12 months, never tested) at each follow-up assessment. Our prespecified secondary outcomes were HIV self-test use, seeking HIV-related medical care, and ART initiation at 1 month and at 4 months. We also assessed adverse events related to HIV self-testing. In addition to the prespecified outcomes, we analyzed 2 additional secondary outcomes (at 1 month and at 4 months): to understand the intervention effects on frequent testing, we assessed testing twice for HIV; and to quantify substitution effects, we assessed facility-based HIV testing. Facility-based testing, i.e., testing in any public or private facility, was determined using a question about the location of HIV testing.

At both follow-up assessments, the research assistants asked participants to self-report the result of their last HIV test (HIV negative, HIV positive, inconclusive, unsure). If participants reported an HIV-positive test result, the research assistants asked them if they sought medical care for their HIV infection and if they were taking antiretroviral medicines. Throughout the study, the research assistants, hotline attendants, and peer educators screened for adverse events related to study participation or HIV self-testing, including physical, sexual, or verbal assault; unintentional HIV status disclosure; and self-harm.

### Sample size

Each study arm received the same number of participants and peer educator groups. We used standard approaches for power calculations for cluster-randomized controlled trials [62]. Assuming 60% HIV testing 1 month following the first peer educator visit in the standard of care arm, 25% loss to follow-up for all participants, type I error probability of 0.05, and an intracluster correlation coefficient (ICC) of 0.02, we estimated that 960 participants in 120 peer educator groups would yield 90% power to detect a risk ratio (RR) of 1.25 in the pooled HIV self-testing arms compared to the standard of care arm. This sample size was also estimated to yield 90% power to detect an RR of 1.18 or larger in the direct provision arm compared to the facility collection arm.

### Statistical analyses

All results are based on intention to treat (ITT) [63–65], complete-case analyses conducted at the unit of the individual with standard errors adjusted for clustering at the peer educator level. Our prespecified analyses were mixed-effect multilevel regression models. We calculated RRs for all binary outcomes using mixed-effects generalized linear models (with Poisson distribution, log link, and robust standard errors) [66], using a fixed effect for study arm and a random effect for peer educator (**S2 Table**). We chose to use modified Poisson models over log-binomial models because they generate similar outcomes and converge more easily when study outcomes are relatively common [66]. We also calculated risk differences for all binary outcomes using the mixed-effects generalized linear models with a fixed effect for study arm and a random effect for peer educator (**S3 Table**). All statistical tests were 2-sided with *p* < 0.05 considered statistically significant.

We conducted 4 sensitivity analyses. First, we pooled the data from the 2 intervention arms and calculated RRs for all outcomes that compared this pooled arm with the standard of care arm using the mixed-effects generalized linear models specified above (**S4 Table**). Second, we calculated the proportion of participants who presented each outcome in a peer educator group. We then used generalized linear models with study arm fixed effects to calculate risk differences for these outcome measures (**S5 Table**). Third, we redefined our outcomes at 4 months to cover shorter time periods than the entire observation period (i.e., the past 1 month, the past 3 months), where such data were available, and calculated RRs using the mixed-effects generalized linear models (described above) (**S6** and **S7 Tables**). Fourth, for the linkage to care outcomes, we limited the sample to participants who reported testing HIV positive and calculated RRs (**S8** and **S9 Tables**). We used Stata 13.1 (StataCorp, College Station, TX) for all analyses.

## Results

### Participants

**Fig 2:**
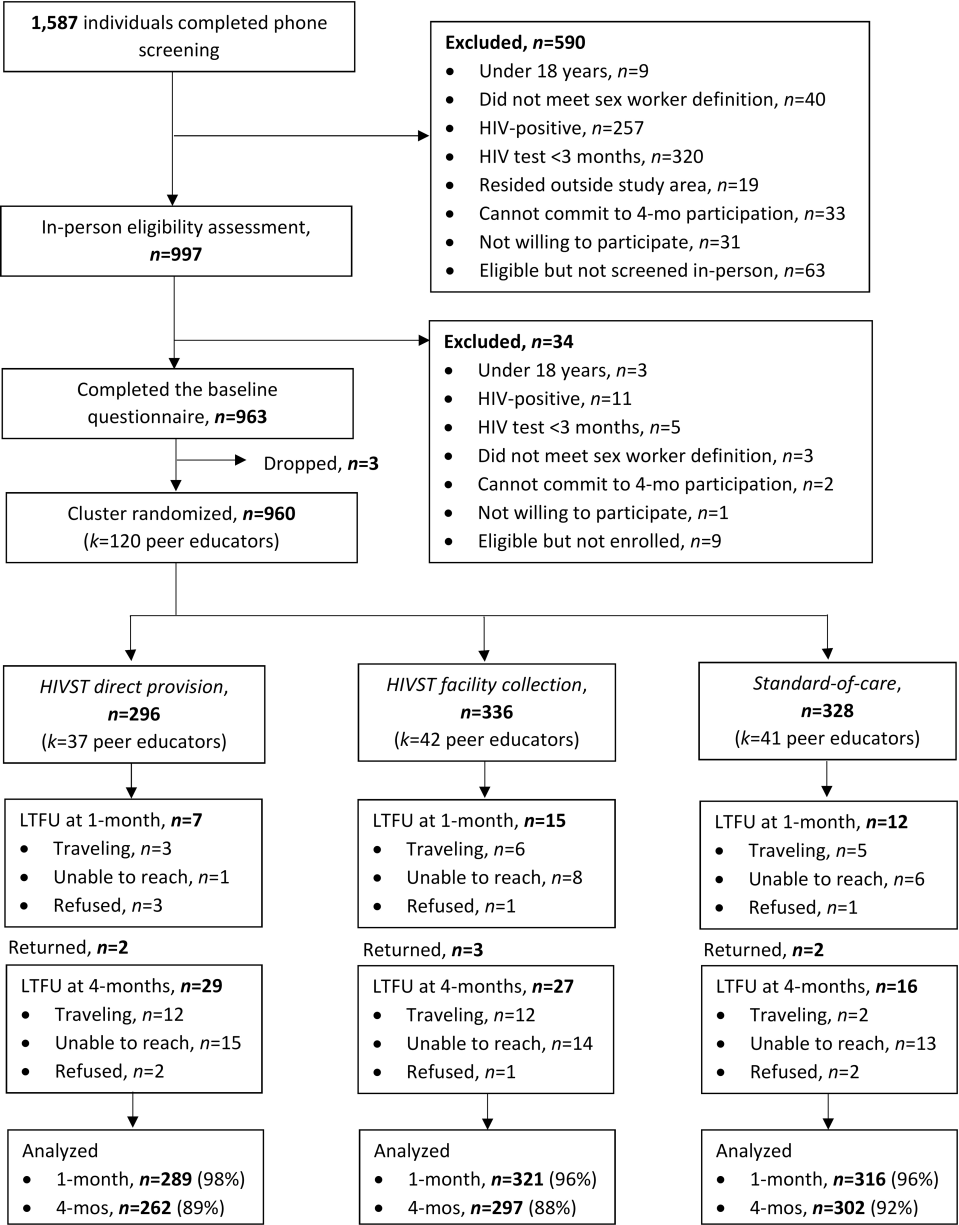
Participant recruitment, eligibility, randomization, and follow-up. CONSORT flow diagram. HIVST, HIV self-test; *n*, number; *k*, clusters; LTFU, loss to follow-up; mo, month.

From October to November 2016, 1,587 potential participants were assessed for eligibility via phone screening, of whom 997 were assessed in person for eligibility and 960 were enrolled in the trial (**Fig 2**). The most common reasons for exclusion from the study were HIV testing within 3 months (325/627, 52%) and self-reported positive HIV status (267/627, 43%). Of the enrolled 960 participants, 296 (31%) were randomized to the direct provision arm, 336 (35%) to the facility collection arm, and 328 (34%) to the standard of care arm. Participant baseline characteristics were balanced across the 3 study arms (**Table 1**). Participant retention at 1 and 4 months was 96.4% (925/960) and 89.7% (861/960), respectively. At 4 months, 18.7% (161/861) of all participants were already members of an FSW peer organization in Kampala. There were no statistically significant differences in loss to follow-up across study arms (**S1 Table**).

**Table 1:** Participant baseline descriptive characteristics.

Characteristic	*Direct provision* (*n* = 296)	*Facility collection* (*n* = 336)	*Standard of care* (*n* = 328)	Total (*n* = 960)
Age (med, IQR)	28 (24–32)	28 (25–32)	28 (24–32)	28 (24–32)
Have primary partner	186 (62.8%)	193 (57.4%)	189 (57.6%)	568 (59.2%)
Can read and write	255 (86.2%)	279 (83.3%)	285 (87.7%)	819 (85.7%)
Education				
*No formal*	24 (8.1%)	35 (10.4%)	20 (6.1%)	79 (8.2%)
*Primary/Junior*	121 (40.9%)	155 (46.1%)	161 (49.1%)	437 (45.5%)
*Secondary*	143 (48.3%)	136 (40.5%)	144 (43.9%)	423 (44.1%)
*Vocational*	2 (0.7%)	6 (1.8%)	0	8 (0.8%)
*Tertiary*	6 (2.0%)	4 (1.2%)	3 (1.0%)	13 (1.4%)
Own mobile phone	289 (97.6%)	311 (92.6%)	310 (94.5%)	910 (94.8%)
Monthly income, PPP-adjusted US dollars^1^				
*No income*	4 (1.4%)	0	1 (0.3%)	5 (0.5%)
*<$105*	63 (21.3%)	76 (22.9%)	51 (15.6%)	190 (19.9%)
*$105 to $218*	90 (30.4%)	117 (35.2%)	125 (38.3%)	332 (34.8%)
*$218 to $436*	104 (35.1%)	107 (32.2%)	117 (35.9%)	328 (34.4%)
*$436 to $873*	31 (10.5%)	25 (7.5%)	29 (8.9%)	85 (8.9%)
*>$873*	4 (1.4%)	7 (2.1%)	3 (0.9%)	14 (1.5%)
Years in sex work (med, IQR)	5 (3 to 8)	5 (3 to 8)	5 (3 to 8)	5 (3 to 8)
Client per night (med, IQR)	5 (4 to 7)	5 (4 to 7)	5 (4 to 7)	5 (4 to 7)
Inconsistent condom use with clients	125 (42.7%)	141 (42.3%)	122 (37.2%)	388 (40.8%)
Timing of last HIV test				
*>3–6 months*	108 (36.7%)	119 (35.6%)	123 (37.5%)	350 (36.6%)
*>6–12 months*	90 (30.6%)	88 (26.4%)	102 (31.1%)	280 (19.3%)
*>12–24 months*	46 (15.7%)	68 (20.4%)	42 (12.8%)	156 (16.3%)
*>24 months*	30 (10.2%)	42 (12.6%)	42 (12.8%)	114 (11.9%)
*Never tested*	20 (6.8%)	17 (5.1%)	19 (5.8%)	56 (5.9%)
Last HIV test facility based^2^	230 (77.7%)	229 (68.2%)	233 (71.0%)	692 (72.1%)
Intimate partner violence, past 3 months				
*Physical*	102 (34.5%)	132 (39.3%)	115 (35.3%)	349 (36.4%)
*Sexual*	89 (30.1%)	105 (31.3%)	94 (28.8%)	288 (30.1%)
*Any*	141 (47.6%)	167 (49.7%)	147 (45.1%)	455 (47.5%)

**Abbreviations:** *n*, total number of participants; IQR, interquartile range; med, median; PPP, purchasing power parity.

^1^Income categories in PPP-adjusted US dollars; World Bank: 1 PPP-adjusted US dollar = 1,146 Ugandan Shillings.

^2^Includes public sector healthcare facilities, private sector healthcare facilities, and antenatal care clinics; other testing locations included: home, work, other.

### Implementation activities

All peer educators reported completing 4 peer educator visits to research assistants over the duration of the study. At 4 months, 88.9% (233/262) of participants in the direct provision arm reported receiving 2 HIV self-tests and 89.9% (267/297) of participants in the facility collection arm reported receiving 2 coupons from their peer educator since the beginning of the study (**Table 2**). Only 4 participants, 2 in the direct provision arm and 2 in the facility collection arm, reported that they did not receive HIV self-tests or coupons from their peer educator. Among participants in the facility collection arm, 72.4% (215/297) reported exchanging 2 coupons for an HIV self-test at a participating healthcare facility and 4.7% (14/297) reported that they did not exchange any coupons for HIV self-tests (**Table 2**). The vast majority of all participants reported receiving condoms at every peer educator visit and there were no statistically significant differences across study arms (**Table 2**).

**Table 2:** Implementation activities reported by participants at 4 months.

Implementation activity	*Direct provision*	*Facility collection*	*Standard of care*	*Total*
Number of HIV self-tests or coupons received by participants				
0	2/262 (0.8%)	2/297 (0.7%)	n/a	4/559 (0.7%)
1	27/262 (10.3%)	28/297 (9.4%)	n/a	55/559 (9.8%)
≥2^1^	233/262 (88.9%)	267/297 (89.9%)	n/a	500/559 (89.5%)
Number of HIV self-tests participants collected at a healthcare facility				
0	n/a	14/297 (4.7%)	n/a	n/a
1	n/a	68/297 (22.9%)	n/a	n/a
2	n/a	215/297 (72.4%)	n/a	n/a
Number of participants who received condoms at every peer educator visit	199/262 (76.0%)	217/297 (73.1%)	232/302 (76.8%)	648/861 (75.3%)

^1^In the direct provision arm, 1.1% (3/262) of participants reported receiving more than 2 HIV self-tests; in the facility collection arm, 0.7% (2/297) of participants reported receiving more than 2 coupons.

### Effects on HIV testing outcomes

**Fig 3:**
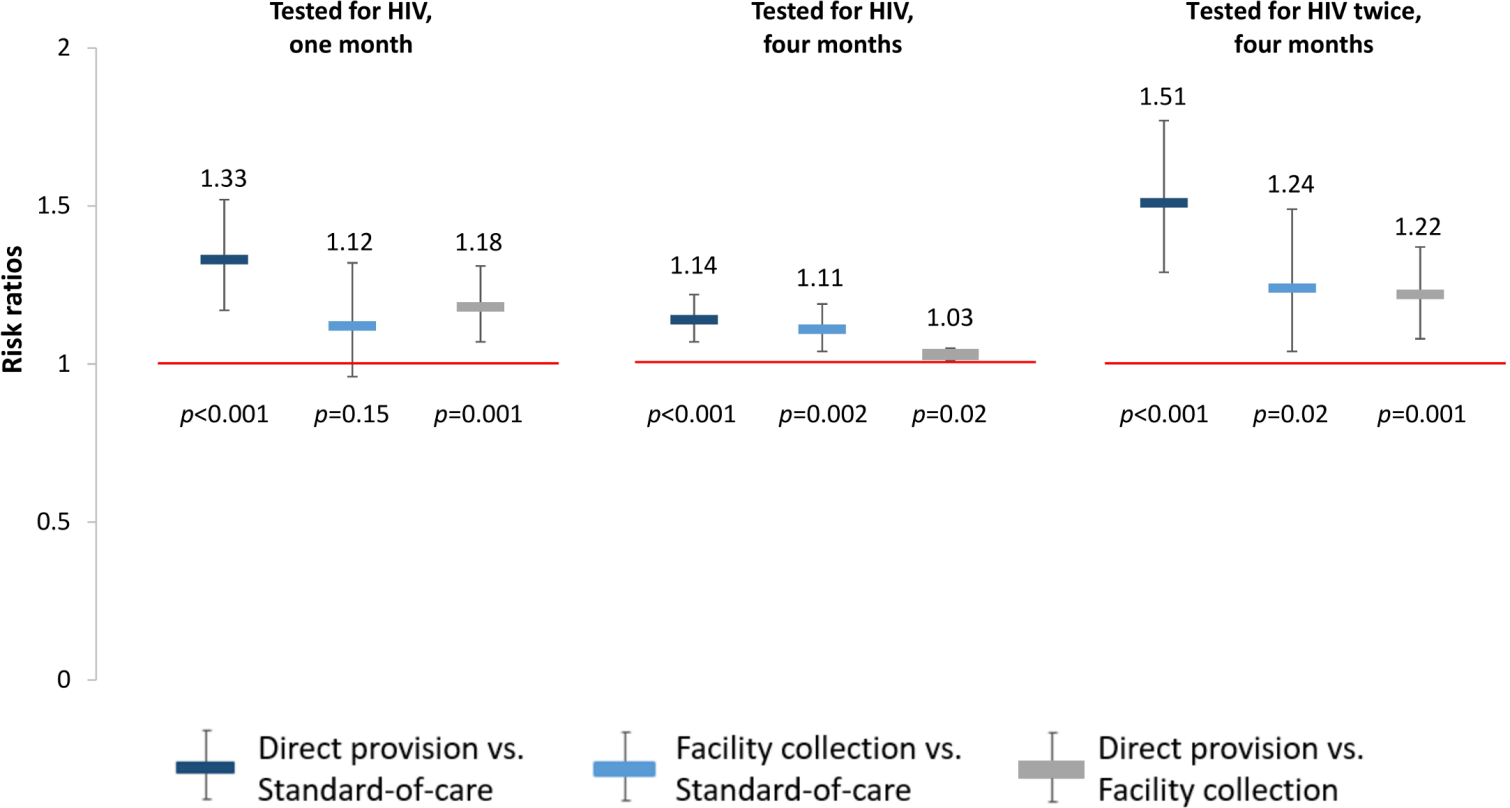
Effect size estimates for impact of HIV self-testing on any HIV testing. All outcomes since study start. Prespecified primary outcomes were any testing at 1 month and at 4 months. Comparisons between study arms: direct provision versus standard of care (dark blue), facility collection versus standard of care (light blue), direct provision versus facility collection (gray).

The effects of HIV self-testing on our prespecified primary outcomes, any HIV testing measured at 1 month and 4 months, were greatest in the direct provision arm, followed by the HIV self-testing facility collection arm and the standard of care arm (“Tested for HIV” in **Table 3**). At 1 month, participants in the direct provision arm were significantly more likely to test for HIV compared to both those in the standard of care arm (RR 1.33, 95% confidence interval [CI] 1.17 to 1.51, *p* < 0.001; risk difference in, percentage points [PP] 24.2, 95% CI 13.9 to 34.5, *p* < 0.001) and those in the facility collection arm (RR 1.18, 95% CI 1.07 to 1.31, *p* = 0.001; PP 14.6, 95% CI 4.4 to 24.9; *p* = 0.005) (“Tested for HIV” in **Fig 3**, **S2** and **S3 Tables**). There were no statistically significant differences in this outcome between participants in the facility collection arm and the standard of care arm (RR 1.12, 95% CI 0.96 to 1.32, *p* = 0.15; PP 9.6, 95% CI −0.4 to 19.6, *p* = 0.06). These effect sizes imply that, compared to the standard of care arm at 1 month, 1 additional participant tested for HIV for every 4 (95% CI 3–7) HIV self-tests (in the direct delivery arm); and, had there been a significant increase in testing at 1 month in the facility collection arm, 1 additional participant could have tested for HIV for every 10 (95% CI 5–250) coupons (in the facility collection arm). The observed ICC for any HIV testing at 1 month was 0.45 (95% CI 0.33–0.58).

**Table 3:** Primary and secondary study outcomes at 1 month and 4 months.

Outcome^1^	1 month			4 months		
	*Direct provision*	*Facility collection*	*Standard-of-care*	*Direct provision*	*Facility collection*	*Standard-of-care*
***HIV testing***						
Tested for HIV*	275/289 (95.2%)	258/321 (80.4%)	226/316 (71.5%)	261/262 (99.6%)	288/297 (97.0%)	263/302 (87.1%)
*Tested for HIV twice*	---	---	---	228/262(87.0%)	212/287 (71.4%)	174/302 (57.6%)
Used an HIV self-test	272/289 (94.1%)	250/321 (77.9%)	0/316 (0%)	258/262 (98.5%)	279/297 (93.9%)	5/302 (1.7%)
*Used a self-test twice*	---	---	---	218/262 (83.2%)	202/297 (68.0%)	---
Tested for HIV at a facility^2^	27/289 (9.3%)	28/321 (8.7%)	211/316 (66.8%)	56/262 (21.4%)	75/297 (25.3%)	259/302 (85.8%)
*Tested for HIV at a facility twice*	---	---	---	4/262 (1.5%)	9/297 (3.0%)	136/302 (45.0%)
Tested HIV-positive	39/287 (13.6%)	54/312 (17.3%)	39/301 (13.0%)	44/260 (16.9%)	80/289 (27.7%)	53/294 (18.0%)
***Linkage to care***^**3**^						
Sought medical care for HIV	17/287 (5.9%)	13/312 (4.2%)	25/301 (8.3%)	27/260 (10.4%)	37/289 (12.8%)	37/294 (12.6%)
Initiated ART	13/287 (4.5%)	10/312 (3.2%)	13/301 (4.3%)	19/260 (7.3%)	27/289 (9.3%)	24/294 (8.2%)

*Prespecified primary outcomes: any HIV testing at 1 month and 4 months.

^1^All testing and linkage to care outcomes self-reported since study start.

^2^Facility-based HIV testing included private and public healthcare facilities.

^3^For these outcomes, participants had to report both testing HIV positive and seeking HIV-related medical care or initiating ART.

At 4 months, participants in the direct provision arm were more likely to test for HIV compared to both those in the standard of care arm (RR 1.14, 95% CI 1.07–1.22, *p* < 0.001; PP 12.9, 95% CI 7.6–18.2, *p* < 0.001) and those in the facility collection arm (RR 1.03, 95% CI 1.01–1.05, *p* = 0.02; PP 2.6, 95% CI −2.7–7.8, *p* = 0.34). Participants in the facility collection arm were significantly more likely to test for HIV at 4 months compared to those in the standard of care arm (RR 1.11, 95% CI 1.04–1.19, *p* = 0.002; PP 10.3, 95% CI 5.2–15.4, *p* < 0.001) (“Tested for HIV” in **Fig 3**, **S2** and **S3 Tables**). These effect sizes imply that, compared to standard of care at 4 months, 1 additional participant tested for HIV for every 8 (95% CI 6–13) HIV self-tests (in the direct delivery arm) and for every 10 (95% CI 7–20) coupons (in the facility collection arm).

**Fig 4:**
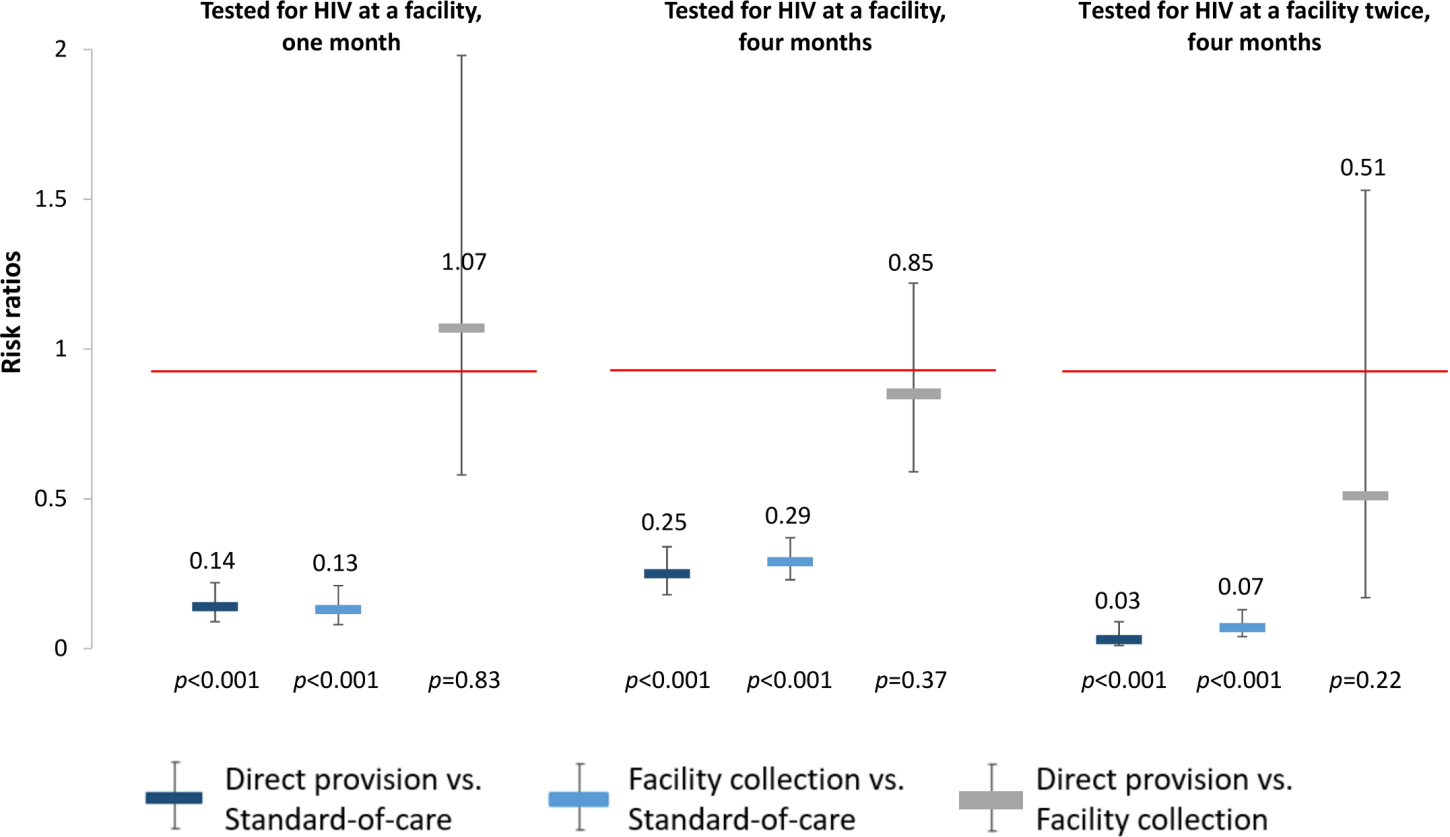
Effect size estimates for impact of HIV self-testing on facility-based HIV testing. All outcomes since study start. Facility-based testing included private and public healthcare facilities. Comparisons between study arms: direct provision versus standard-of-care (dark blue), facility collection versus standard-of-care (light blue), direct provision versus facility collection (gray).

To better understand the effects of the HIV self-testing interventions, we also determined the effect of the interventions on repeated HIV testing. At 4 months, participants in both self-testing intervention arms were significantly more likely to have tested for HIV twice compared to those in the standard of care arm (direct provision RR 1.51, 95% CI 1.29–1.77, *p* < 0.001; facility collection RR 1.24, 95% CI 1.04–1.49, *p* = 0.02), and participants in the direct provision arm were significantly more likely to have tested for HIV twice compared to those in the facility collection arm (RR 1.22, 95% CI 1.08–1.37, *p* = 0.001) (“Tested for HIV twice” in **Fig 3**, **S2** and **S3 Tables**). Finally, to better understand the relationship between the availability of the HIV self-testing technology and HIV testing outcomes, we additionally assessed the effect of the HIV self-testing interventions on facility-based testing. Facility-based testing was significantly lower among participants in the HIV self-testing intervention arms compared to the standard of care arm, both at 1 month (direct provision RR 0.14, 95% CI 0.09–0.22, *p* < 0.001; facility collection RR 0.13, 95% CI 0.08–0.21, p < 0.001) and at 4 months (direct provision RR 0.25, 95% CI 0.18–0.34, *p* < 0.001; facility collection RR 0.29, 95% CI: 0.23–0.37, *p* < 0.001), (“Tested for HIV at a facility” in **Fig 4**, **S2** and **S3 Tables**). In the standard of care arm, private facility-based testing was more common than public facility-based testing at both 1 month (private 40.5%, 128/316; public 26.6%, 83/316) and 4 months (private 56.6%, 171/302; public 31.8%, 96/302) (**S10** and **S11 Tables**).

### Effects on linkage to care outcomes

Linkage to care outcomes were low at both 1 month and 4 months (“Sought medical care for HIV” and “Initiated ART” in **Table 3**). Overall, few people tested HIV positive and were eligible for linkage to care (because FSWs with known positive HIV status were excluded from our study). Seeking HIV-related medical care appeared to be lower in both the HIV self-testing intervention arms compared to the standard of care arm at 1 month (direct provision RR 0.65, 95% CI 0.30–1.41, *p* = 0.28; facility collection RR 0.50, 95% CI 0.24–1.04, *p* = 0.06), but these differences were not statistically significant and were reduced in magnitude at 4 months (“Sought medical care for HIV” in **Table 3** and in **S2** and **S3 Tables**). There were no statistically significant differences in ART initiation across study arms (“Initiated ART” in **Table 3** and in **S2** and **S3 Tables**). However, the CIs around the linkage to care effect point estimates are very wide, indicating a high degree of uncertainty regarding this finding.

### Sensitivity analyses

All of our sensitivity analyses confirmed our main analyses. In the analysis pooling the data from the 2 HIV self-testing intervention arms, any HIV testing was significantly higher in the pooled arm compared to the standard of care arm at both 1 month (RR 1.22, 95% CI 1.07–1.40, *p* = 0.004) and 4 months (RR 1.13, 95% CI 1.05–1.21, *p* < 0.001) (“Tested for HIV” in **S4 Table**). In the analysis using the proportion of participants in a peer educator group, the outcome any HIV testing was significantly higher in the direct provision arm than in the standard of care arm at both 1 month (PP 24.4, 95% CI 14.1–34.7, *p* < 0.001) and 4 months (PP 13.1, 95% CI 7.9–18.4, *p* < 0.01) (“Tested for HIV” in **S5 Table**). In the sensitivity analyses using outcomes at 4 months that covered shorter time periods than the entire observation period, any HIV testing in the past month was significantly higher in the direct provision arm (RR 1.35, 95% CI 1.08–1.69, *p* = 0.009) and the facility collection arm (RR 1.29, 95% CI 1.00–1.66, *p* = 0.05) compared to the standard of care arm (“Tested for HIV (past month)” in **S6** and **S7 Tables**). All of the other ITT sensitivity analyses also demonstrated robustness of the results described above (**S4**–**S7 Tables**).

In the linkage to care sensitivity analysis, which was conditioned on participants who reported testing HIV positive, seeking HIV-related medical care was again lower in the HIV self-testing intervention arms compared to the standard of care arm, but now this difference among participants in the facility collection arm was statistically significant at both 1 month (RR 0.38, 95% CI 0.21–0.67, *p* = 0.001) and 4 months (RR 0.66, 95% CI 0.47–0.94, *p* = 0.02) (“Sought medical care for HIV” in **S8 Table**). There were no statistically significant differences in ART initiation across study arms in this analysis, confirming the results from the ITT analysis (“Initiated ART” in **S8 Table**). Limiting the sample size to participants who reported testing HIV positive post-randomization could have introduced selection biases. In as far as we can test such biases through balance tests of observed participants’ baseline characteristics across study arms, any selection biases seem unlikely to be severe. We only found a statistically significant lack of balance in one of the characteristics we tested (mobile phone ownership, **S9 Table**). Additionally, the significance of linkage findings remained robust in models that adjusted for participant’s age, highest level of education, and monthly income (**S8 Table**).

### Adverse events

Adverse events were rare; 4 were reported over the duration of the study. Two were related to HIV self-testing: (1) interpersonal violence (verbal abuse from boyfriend, in the facility collection arm) and (2) mental distress following a positive HIV self-test result (the participant later tested HIV negative at a healthcare facility, in the direct provision arm). The additional 2 adverse events were interpersonal violence related to FSW status disclosure. Use of the study hotline was uncommon; only 7% (59/861) of participants called the hotline by 4 months and only 8 (14%) of these calls were for reasons related to HIV self-testing (questions regarding HIV self-test use or results interpretation).

## Discussion

We find that oral HIV self-testing is safe and effective at increasing HIV testing among FSWs. In our study done in Kampala, Uganda, the provision of HIV self-tests significantly increased the likelihood that FSWs participated in HIV testing at 1 month and additionally resulted in almost universal HIV testing at 4 months. Within a 4-month period, FSWs in the HIV self-testing arms were also significantly more likely to test twice for HIV compared to those in the standard of care arm. Universal and repeated HIV testing is particularly important for FSWs because of the high risk of HIV acquisition they face in their daily lives [14,15].

For their own health, frequent HIV testing will allow FSWs to detect HIV infection early and initiate treatment with minimal delay. Frequently repeated HIV testing is also a prerequisite for PrEP, which is becoming increasingly available to FSWs in sub-Saharan Africa. PrEP requires frequent HIV testing to detect breakthrough infections [13]. Our results suggest that HIV self-testing could be an important approach to ensure that FSWs who are taking PrEP regularly check their HIV status [67]. Of course, the viability of combining PrEP and HIV self-testing will depend on further investigation of the performance of the HIV self-tests in detecting HIV among PrEP users [68].

For the health of others, frequent HIV testing is necessary for successful TasP and positive prevention strategies [11,12]. FSWs have larger numbers of sex partners than most other population groups [15] and are thus at increased risk of spreading the virus following infection [14–16]. Frequent HIV testing will ensure early detection of infection, which is needed for early treatment initiation and behavior change to prevent onward HIV transmission. Our findings regarding the effect of HIV self-testing on frequent testing thus suggests that self-testing could play an important role in reducing transmission risk among FSWs.

While our findings indicate that HIV self-testing is effective overall in increasing recent and repeat HIV testing, the effectiveness of one of the two delivery models that we tested—direct provision of HIV self-tests—was better than that of the other model—facility collection of HIV self-tests. Direct provision of HIV self-testing eliminates more potential barriers to HIV self-testing than facility collection, requiring neither an interaction with a health worker nor money or time for travel to test for HIV. In contrast, facility collection of HIV self-tests requires some interaction with health workers, who might stigmatize FSWs. Moreover, to collect an HIV self-test in a healthcare facility, an FSW needs to spend amounts of time and money similar to the amounts necessary for facility-based testing.

We included the facility collection arm in our study because it closely resembles the likely default strategy to HIV self-testing that governments in sub-Saharan Africa will choose. In fact, in South Africa [69] and Kenya [70], HIV self-tests have already become available for over-the-counter purchase in pharmacies [71]. Our results show that for FSWs, such passive provision of HIV self-tests in facilities is inferior to the active delivery of HIV self-tests through peer educators. In adopting HIV self-testing policies, governments in sub-Saharan Africa should consider peer-supported strategies of direct HIV self-test delivery for FSWs as well as for other key populations that are likely to face healthcare provider stigma and lack the time and money for frequent travel to healthcare facilities.

Another important secondary finding of our study is that the HIV self-testing interventions not only increase overall HIV testing but also lead to a very high degree of substitution of facility-based testing with self-testing. At 1 month, less than 10% of all testing was facility based in the self-testing intervention arms, while more than 60% of testing was facility based in the standard of care arm; at 4 months, about one-fourth of all testing was facility based in the self-testing arms, while more than 80% was facility-based in the standard of care arm. This substitution has several important implications. First, it signals a high degree of acceptance of HIV self-testing among FSWs in Uganda, which bodes well for future routine government rollout of HIV self-testing strategies in the country. Second, in the direct provision arm, the large substitution effect implies substantial money and time savings for FSWs compared to facility-based HIV testing. These savings are an additional benefit of direct provision of HIV self-tests and are particularly important because FSWs are typically very poor [15]. Third, HIV self-testing moves HIV testing outside the regulated environment of the health system. Here, correct HIV status knowledge depends on the tester's correct interpretation of the self-test results. Previous studies have found high sensitivity and specificity of self-interpreted HIV self-test results [43,44,72,73], but none of these studies were conducted among FSWs. The overall value of HIV self-testing as an approach to increase recent and frequent HIV testing among FSWs will critically depend on ensuring that FSWs have the skills to correctly interpret the self-test results.

The large substitution effects also raise the worry of potential negative consequences for linkage to care. Self-testing will typically take place outside a healthcare facility and often far from the closest facility where HIV treatment and other services are available. Moreover, self-testing will generate an HIV test result without accompanying pre- and post-test counselling by a specifically trained health worker, as is the standard in facility-based testing. Both of these characteristics of self-testing could decrease linkage to care. Our linkage to care analyses suggest that this may be an important concern. In both the ITT analyses and the sensitivity analyses estimating linkage effects only among those FSWs who reported that they tested HIV positive, the percentage of participants who reported seeking HIV-related medical care at 1 month was lower in the HIV self-testing intervention arms compared to the standard of care arms. This difference was not statistically significant and disappeared by 4 months in the ITT analyses, but it was statistically significant and remained so at 4 months in the conditional sensitivity analyses.

It is important to note in this regard that the sensitivity analyses estimating linkage effects of HIV self-testing only among those FSWs who reported that they tested HIV positive will likely be biased. Randomization only ensures that we are comparing “like” and “like” in ITT analysis; in contrast, analyses that are conditional on events that occur after randomization can suffer from selection biases. One such type of event is the process that leads to HIV testing and reporting of a positive HIV test result. The intervention itself could influence this selection process, leading to biased effect size estimation when outcomes are conditional on participants reporting positive HIV status, as is the case for the linkage analysis conditional on positive status. For instance, the option to test oneself for HIV may induce some FSWs who would never test in a facility to test for HIV for the first time. These FSWs will be in the denominator of persons who are HIV-positive in the intervention arms, but they will not be in the same denominator in the standard of care arm. If the characteristics that prevented these FSWs from testing in healthcare facilities—e.g., fear of provider stigma—also prevents them from linking to HIV care, the effect size estimation in the conditional analysis will be biased.

Future studies are needed to provide stronger evidence of the impact of HIV self-testing on linkage. These studies will require substantial investments. Compared to previous studies [46–49], in our study a large number of people (a total of 177 across the 3 arms) tested HIV positive and were thus eligible for linkage. Nevertheless, eligibility for linkage was still comparatively rare among study participants and our study lacked sufficient power for strong conclusions on the effects of HIV self-testing on linkage to care. Until better evidence becomes available, HIV self-testing policies for FSWs should ideally include strong linkage interventions. Such interventions are available. Successful examples of linkage-enhancing interventions include counseling by peer educators [74,75], home- and community-based ART [76,77], and financial incentives [78,79].

Our results indicate that FSWs preferred the direct provision of HIV self-tests by peer educators over access to HIV self-testing via a coupon that could be exchanged for an HIV self-test in healthcare facilities. It is possible that this preference is due to the time and monetary costs incurred when traveling to a facility to collect an HIV self-test. In future research, it would be interesting to compare peer-provided HIV rapid testing with peer-provided HIV self-tests. Both of these HIV testing modalities eliminate the costs of traveling to a healthcare facility. However, peer-provided HIV self-tests differ from peer-provided HIV rapid testing in several ways: the former strongly protects the privacy of test results, while the latter implies disclosure of results to a peer; the former allows FSWs to mentally prepare for an HIV test and to test at the preferred time and location, while the latter requires HIV testing when and where the peer educator is available. Our 3-arm comparison allows some inferences regarding the testing preferences relevant to judge these 2 alternative testing options. At the time of our study, there were several HIV testing initiatives targeted to FSWs in Kampala, including home- and work-based HIV testing. The fact that FSWs in both intervention arms almost exclusively used HIV self-tests instead of other HIV testing options suggests that FSWs highly valued the distinguishing characteristics of HIV self-tests (strong privacy protection, unconstrained choice of testing time and location), independent of the delivery model used to provide the self-test. It thus seems likely that many FSWs will prefer peer-provided HIV self-testing over peer-provided HIV rapid testing. At the same time, the difference in the effects of the direct provision and the facility collection approaches to HIV self-testing was likely caused by the different costs associated with these 2 delivery models. It therefore seems likely that many FSWs will prefer peer-provided HIV rapid testing over facility collection of HIV self-tests.

Our study has several important strengths, including the testing of 2 different HIV self-testing delivery models, the large sample size, the low loss to follow-up, and the exclusive focus on FSWs. Our study also has important limitations. For one, we rely on self-reported outcomes, which could be biased by social desirability and other reporting distortions. For instance, participants in the direct provision arm might have felt more shame for not using an HIV self-test than those in the facility collection arm, because they received an HIV self-test directly from their peer educators and did not have to invest time and money to obtain a self-test in a facility. The participants in the direct provision arm might thus have felt more pressure to over-report HIV testing and self-test use.

In addition, the external validity of our results may be limited. First, all participants in our study received the peer educator intervention, which included condom distribution, information about HIV testing options, and encouragements to test for HIV. Thus, we were unable to measure the effect of HIV self-testing in the absence of these peer educator activities. Second, the peer educators delivering the interventions in our trial reached out to fellow sex workers within their social networks. This recruitment process might further threaten the external validity of our findings. For instance, peer educators may have selected those FSWs who they knew would be interested in exploring new HIV testing options. Third, FSWs in urban Kampala are also highly organized and benefit from ongoing policy initiatives by the Ugandan government aimed at providing HIV services to FSWs. The high background levels of HIV testing in our study population are likely a result of these services. Our findings may thus not generalize to FSWs in settings with fewer HIV testing interventions targeting FSWs. Fourth, FSWs enrolled in this study were recognized as such by their peers. Sex work, however, manifests in a variety of different forms, and peer-based HIV self-testing delivery models might have different effects in less formal FSW populations, such as girls who date sugar daddies and barmaids who only occasionally sell sex.

In sum, oral HIV self-testing could be an important component of HIV policies to achieve near-universal and frequent HIV testing among FSWs. In particular, HIV self-testing could support HIV interventions that require frequent HIV testing, such as TasP, behavior change for transmission reduction, and PrEP. In designing HIV self-testing policies for FSWs, governments should consider direct provision of HIV self-tests rather than merely making HIV self-tests available in healthcare facilities. HIV self-testing policies for FSWs should be accompanied by strong interventions to support linkage to care.

## Supporting information

S1 Text Trial protocol—Manual of Operations. Our trial protocol, including prespecified primary and secondary outcomes, can be found at ClinicalTrials.gov (under the registration number NCT02846402). Provided here is our Manual of Operations.
(DOCX)

S2 Text CONSORT statement. (DOC)

S1 Table Loss to follow-up and refusal to answer questions about HIV status at 1 month and at 4 months. (DOCX)

S2 Table Effect size estimates: Risk ratios. RR, risk ratio.
(DOCX)

S3 Table Effect size estimates: Risk differences. PP, percentage point.
(DOCX)

S4 Table Sensitivity analysis: Pooled HIV self-testing arms versus the standard of care arm. RR, risk ratio.
(DOCX)

S5 Table Sensitivity analysis: Proportion of participants in a peer educator group reporting each outcome. PP, percentage point.
(DOCX)

S6 Table Noncumulative outcomes at 4 months. (DOCX)

S7 Table Sensitivity analysis: Noncumulative outcomes at 4 months. RR, risk ratio.
(DOCX)

S8 Table Sensitivity analysis: Linkage to care among participants who reported testing HIV positive. RR, risk ratio.
(DOCX)

S9 Table Baseline descriptive characteristics for participants who reported testing HIV positive at 4 months. (DOCX)

S10 Table Private and public healthcare facility-based testing at 1 month and at 4 months. (DOCX)

S11 Table Effect size estimates: public and private healthcare facility-based testing. RR, risk ratio.
(DOCX)

## Abbreviations

CI: confidence interval
FSW: female sex worker
ICC: intracluster correlation coefficient
ITT: intention-to-treat
IPV: intimate partner violence
MARPI: Most at Risk Populations Initiative
MOH: Ministry of Health
NGO: nongovernmental organization
PP: percentage point; PPP, purchasing power parity
PrEP: pre-exposure prophylaxis
RR: risk ratio
TasP: treatment as prevention
