# Supplementary material for: Direct provision versus facility collection of HIV self-tests among female sex workers in Uganda: A cluster-randomized controlled health systems trial
Source: PLoS Med. 2017 Nov 28;14(11):e1002458. doi: 10.1371/journal.pmed.1002458 (PMC5705079; doi:10.1371/journal.pmed.1002458)
Supplement: S1 Text — Our trial protocol, including prespecified primary and secondary outcomes, can be found at ClinicalTrials.gov (under the registration number NCT02846402). Provided here is our Manual of Operations. (DOCX) [file pmed.1002458.s001.docx]

**The Causal Impact of HIV Self-Test Kits on HIV Testing Among FSW in Kampala, Uganda (HSPOT)**

**Manual of Operations and Procedures (MOP)**

**Participating Organizations**

Uganda Health Marketing Group, Kampala, Uganda

International Research Consortium, Kampala, Uganda

Harvard T.H. Chan School of Public Health, Boston, MA, USA

**Investigators**

Daniel Kibuuka Musoke, MBChB, MSc

Thomson Ngabirano, MBChB, MPH

Jonathan Magoola, MSc

Prossy Nampala Walusimbi Kayira, MScPH, BACE, RN

Katrina Ortblad, MPH

Catherine Oldenburg, MPH, ScD

Guy Harling, MA, ScD

Till Bärnighausen, MD, ScD

**TABLE OF CONTENTS**

**1. Introduction** …………………………………………………………………………. 4

**2. Background and Significance** ………………………………………………………. 5

**3. Organization and Policies** …..………………………………………………………. 8

**4. Participant Flow** ……………..……………………………………………………... 14

**5. HIV Self-Testing Kits**……………..………………………………………………... 22

**6. Intervention Components** ………..………………………….……………………... 23

**7. Key Informant (Peer Educator) Focus Groups** …………………………………... 24

**8. Accuracy Study**………......………..………………………………………………... 25

**9. Outcome Assessment**….…………..………………………………………………... 27

**10. Protection of Human Subjects**…………..………………………………………... 32

**11. Data Management**………………..………………………………………………... 36

**12. Statistical Analysis**…...…………..………………………………………………... 38

**13. Appendix Outline**..…...…………..………………………………………………... 40

**14. References** ………………………..………………………………………………... 42

**1. INTRODUCTION**

More than one in three female sex workers (FSW) are estimated to live with HIV in Southern Africa.^[[1]](#endnote-1)^ HIV vulnerability in this key population is affected by multiple factors, including structural factors (i.e., economic and healthcare marginalization, punitive laws^[[2]](#endnote-2)^), interpersonal factors (economic dependence on clients, vulnerability to violence^[[3]](#endnote-3)^), and individual factors (condom use with intimate and economic partners).^[[4]](#endnote-4)^ Stigma and discrimination, in particular, act as a barrier for FSW to access HIV-related services, including counseling and testing. HIV self-testing is an alternative HIV testing modality that may be more acceptable to FSW, given that it can be done in private and during a time that is convenient for the woman testing.

To assess whether HIV self-testing is a viable option for FSW in Uganda, we propose a pilot intervention and impact evaluation to establish a preferred distribution mechanism of HIV self-tests for FSWs in Kampala. HIV self-tests will be distributed via two distinct mechanisms: 1) *fixed distribution points* (i.e., participating Good Life Clinics) following referral from peer educators, and 2) *direct distribution* from peer educators. Peer educators will be assigned to specific FSW to minimize contamination and facilitate outcome assessment. We propose a cluster-randomized trial in which peer educators are randomized to one of three arms, including each distribution mechanism (fixed distribution, direct distribution) as well as referral to standard HIV testing mechanisms (standard of care).

To assess the effects of this HIV self-testing intervention, we propose the following specific aims:

**Aim 1: Establish the causal impact of the intervention on rates of recent HIV testing among FSWs surrounding HIV hotspots.** We hypothesize that HIVST intervention will increase rates of recent HIV testing among FSWs.

**Aim 2: Determine the causal impact the intervention has on knowledge of HIV status among FSWs surrounding HIV hotspots.** We hypothesize that the HIVST intervention will increase knowledge of HIV status increases among FSWs.

**Aim 3: Understand how the intervention influences individuals’ sense of empowerment surrounding their ability to protect themselves from infection and control sexual interactions.** We hypothesize that the HIVST intervention empowers FSWs to demand the use of HIV prevention measures with their sexual partners.

Embedded within this study we will also be conducting an oral HIVST kit accuracy study among willing peer educators and we will collect cost data so we can evaluate the cost effectiveness of oral HIV self-testing between our two treatment groups.

**2. BACKGROUND AND SIGNIFICANCE**

**While HIV prevalence among the general population in sub-Saharan Africa is higher than anywhere else in the world, female sex workers (FSWs) continue to be at much higher risk of HIV infection risk than the general population.** In 2014, roughly 37 million people were living with HIV; of these 37 million, 19 million (52% globally) live in East and Southern Africa.^[[5]](#endnote-5)^ In Uganda alone, 1.5 million individuals are living with HIV/AIDS; which is roughly 7.3% of the population.^[[6]](#endnote-6)^5,6 Estimates of national HIV prevalence in East and Southern African countries ranges from 1.1% in Burundi to 27.7% in Swaziland.^5^ These percentages are high, but evidence shows that HIV prevalence among FSW is often 12 times greater than the general population.^5^ A pooled analysis of 16 sub-Saharan African countries found HIV prevalence to be around 37% in FSWs in 2012;^5^ the prevalence of HIV among FSWs in Uganda has been estimated at 33%.^[[7]](#endnote-7)^7

**HIV antiretroviral treatment is highly successful in ensuring that individuals diagnosed with HIV/AIDS can live full, healthy lives and do not transmit HIV to others – but HIV diagnosis and linkage to care are necessary pre-conditions for these benefits.** HIV is no longer the death sentence it once was in the past; high quality antiretroviral therapy (ART) not only keeps individuals alive, but has also been shown to prevent the spread of HIV infection.^[[8]](#endnote-8)^8^,^^[[9]](#endnote-9)^9 HIV-positive individuals who adhere to treatment can reduce their viral load and also reduce the probability of spreading the virus to their HIV-negative partner(s). Many FSWs continue to work after HIV infection, thus identifying these individual and linking them to care can be an effective HIV prevention strategy. International donations as well as governmental investments have made ARTs free of cost in most developing and developed countries.^[[10]](#endnote-10)^10 Despite the availability of these drugs, individuals around the world continue to die from HIV because they are lost somewhere along the HIV treatment cascade.^[[11]](#endnote-11)^11 The treatment cascade begins when an individual is infected with HIV then goes through the necessary stages of the health system that ensure this individual is virally suppressed (<200 copies/mL). These stages include HIV diagnosis, linkage to care, retention in care, ART initiation, and proper ART use. Unfortunately, individuals are lost at each stage of this cascade. In sub-Saharan Africa it is estimated that roughly 40% of HIV-positive individuals are never diagnosed with HIV and of positively diagnosed individuals, 59% are not linked to care.^[[12]](#endnote-12)^12,^[[13]](#endnote-13)^13,^[[14]](#endnote-14)^14 With such large gaps early on in the cascade, the lifesaving effects of high quality treatment are limited.

**Recognizing that these gaps in the HIV treatment cascade are critical impediments to realizing the potential benefits of ART, UNAIDS is committed to a new global target for 2020, the 90-90-90 target.** The 90-90-90 target aims to have 90% of all people living with HIV to know their status, 90% of all people with diagnosed HIV infection to received sustained antiretroviral therapy, and 90% of people receiving antiretroviral therapy to have viral suppression by 2020.^[[15]](#endnote-15)^15 New strategies to increase HIV testing include voluntary and provider-initiated counseling and testing as well as mobile testing and home-based testing. In Uganda, despite a 14% to 60% rise in HIV counseling and testing (HCT) among individuals aged 15-49 from 2004 to 2012 as a result of these new strategies, HIV testing remains far from UNAIDS 90% target.^[[16]](#endnote-16)^16

**Female sex workers often have limited accessibility and uptake of health services (in particular HIV prevention, treatment, and care) as a result stigma and discrimination, violence and punitive legal and social environments.^8^** Violence among FSWs is common and often the perpetrators of this violence are law enforcement officials themselves. This, in addition to the fact that their work is often illegal, makes it difficult for FSWs to report this violence and trust government services. Fear of arrest or brutality makes FSWs highly mobile populations, making it difficult for them to have a regular health care provider and familiarize themselves with health services in their area. Many FSWs have also been denied health services as a result of stigma and discrimination.^5^ A 2009 Uganda study found that three quarters of FSWs reported they did not have access to family planning or contraception, indicating their low uptake of and access to health services.^7,^^[[17]](#endnote-17)^17 Because many FSWs don’t have access to HIV prevention services and many of them are at risk of sexual violence, they have an increased risk of HIV infection. Consequently, it is important that this population test for HIV on a regular basis so they can protect both themselves and their sexual partners from HIV transmission.

**Oral HIV self-testing (HIVST) has the potential to remove existing barriers to HCT and increase rates of both HIV testing and linkage to care.** Since oral HIVST can be done in the privacy of one’s home, it allows individuals who are wary of the health system or do not have time in their schedules to visit health facilities to test for HIV. HIVST is the one HIV testing technology that is controlled by the tester, thus empowering vulnerable populations, such as FSWs, by providing ways to safely access health services and avoid violence, stigma, and discrimination. HIV status knowledge can decrease fears surrounding HIV risk acquisition and increase bargaining power of barrier methods at time of sex. If more HIV-positive individuals are aware of their status as a result of HIVST, they might also be incentivized to access care; increasing the number of individuals linked to HIV services. HIVST is a cheaper testing strategy than clinic- and home-based HCT, especially if existing systems like moonlight clinics are used for distribution. This may make HIVST cost-effective for national health systems to implement and ideal for frequent testers (e.g. FSWs). HIVST feasibility studies conducted in Kenya and Zambia have found near universal interest in oral HIVST,^[[18]](#endnote-18)^18 however, in a number of countries HIVST remains illegal or no formal policy on HIVST exists.^[[19]](#endnote-19)^19 Fears around HIVST include unregulated kits, improper use, social and emotional harm following test, coercion to test, and poor linkage to care,^[[20]](#endnote-20)^20 but little evidence exists to validate these concerns.^[[21]](#endnote-21)^21 Studies on the impact of HIVST on overall HIV testing rates and linkage to care are currently of the utmost importance so global guidelines can be formulated surrounding their use.

**Peer educator networks are widely used in HIV prevention programs across population groups and geographical areas.** Review of some of the studies that have evaluated HIV/AIDS peer education programs using experimental or quasi-experimental designs, with outcome indicators such as reduction of HIV-related risk behavior and/or STI/HIV incidence, shows that peer education (in combination with other prevention strategies) is very effective in several populations and geographical areas including key populations. Engaging peer educators in HIV prevention activities leads to significant changes in knowledge, attitudes, and self-efficacy among FSW. In order to achieve best results, project should select peer educators with the interest/ motivation to participate in project activities, in addition to training, supervising and providing proper incentives.^[[22]](#endnote-22)^22

**HIV testing coverage remains suboptimal in much of the world, particularly among key populations such as female sex workers (FSW).** Correct knowledge of one’s HIV status is essential for timely linkage to care and initiation of treatment, the first step in the UNAIDS 90-90-90 target. FSW are a group that benefits from regular HIV testing, however they face substantial barriers to regular testing. HIV self-testing is an alternative to clinic or other provider-based HIV testing that allows individuals to test in the privacy of their own homes and on a time schedule that is most beneficial to them. As the test is performed by an individual in private without relying on a healthcare provider, it is also confidential. The ease of use of HIV self-testing, that it can be done any time, and that it is completely private may make it an attractive alternative to currently-available HIV testing mechanism for FSW in Zambia.

**HIV self-testing has generally been shown to be acceptable to a variety of populations, however evidence related to its uptake and use remains sparse, particularly among key populations and in Sub-Saharan Africa.** This research will provide rigorous evidence of the uptake and efficacy of HIV self-testing for this population. The evidence generated by this study is intended to guide policy in Uganda on distribution of HIV self-tests among FSW.

**3. ORGANIZATION AND POLICIES**

**3.1 Study Organization**

**3.1.1 Implementation Team**

The Implementation Team will be led by Dr. Thomson Ngabirano of UHMG, Kampala. This team will oversee the implementation of the HIV self-testing intervention. The team will meet regularly and will make decisions on day-to-day operations issues. The implementation will meet on a weekly basis with the evaluation team. The implementation team will oversee the following:

- Training for peer educators
- Work with Kampala Good Life Clinics on implementation of the project
- Procure HIV self-tests for implementation
- Monitor implementation progress and recruitment
- Oversee peer educators, drug store, and health facility staff throughout course of project to ensure fidelity to protocols
- Make executive decisions on the allocation of resources
- Evaluation of adverse events that arise during the study

**3.1.2 Evaluation Team**

The Evaluation Team will be led by Dr. Till Bärnighausen at the Harvard School of Public Health in Boston with close collaboration with Dr. Daniel Kibuuka Musoke at the International Research Consortium (IRC) in Kampala, who will serve as the Ugandan PI for this study. This team will oversee the evaluation of the project. The team will meet on a weekly basis and will also meet weekly with the implementation team. The evaluation team will oversee the following:

- Training of research staff in collaboration with the implementation team
- Design of study tools for data collection
- Quality control for data collection
- Generation of randomization lists
- Coordination of Institutional Review Board approvals
- Evaluation of adverse events that arise during the study

**3.1.3 Scientific Oversight Committee**

The Scientific Oversight Committee will consist of independent experts in clinical trials, HIV epidemiology, and key populations from both Uganda and the United States. The Committee will be empaneled prior to the beginning of the study and will meet at the beginning of the study and mid-way through the meeting via teleconference. Ad hoc meetings as needed may also be convened. All study protocols will be reviewed by the appropriate Institutional Review Boards in addition to the Scientific Oversight Committee. Committee members will review any adverse events that occur during the course of the study that threatens the safety of participants.

| **Agency** | **Representative** |
| --- | --- |
| **MOH** | 1. Dr Joshua Musinguzi 2. Dr Kirungi Wilford 3. Dr Shaban Mugerwa 4. Geoffrey Taasi 5. Dr Peter Kyambadde 6. Michael Muyonga 7. Dr Linda Nabitaka 8. Walimbwa 9. Richard Kabagambe |
| **CT 17 & AIDS Development Partners (ADPS)** | 1. Rita Nalwadda CDC 2. Madina Apolot WHO 3. Rosemary Kidyomunda UNFPA |
| **UVRI**  **CPHL**  **UNHCO** | 1. **UVRI - tbd** 2. Michael Nfendu 3. Robinah Kaitiritimba |

**3.2 Organizational Management Plan**

**3.2.1 Overall Organization**


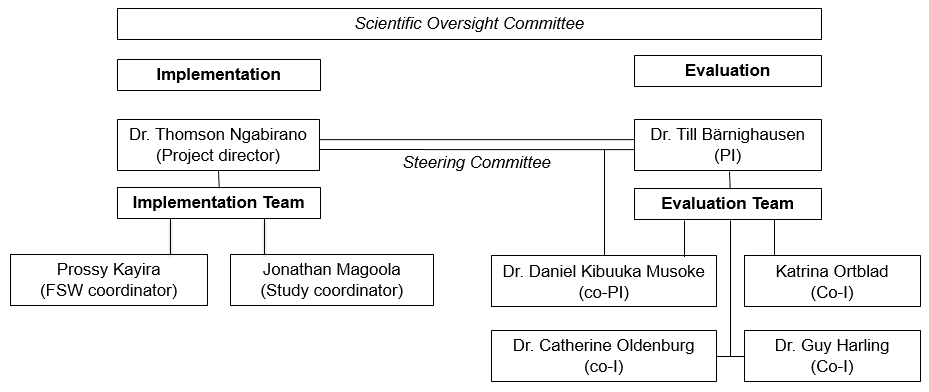


***Figure 1.*** *Overall organization of the HSPOT study.*

The HSPOT project is organized into two distinctive teams, a Kampala-based implementation team and a Kampala-Uganda based evaluation team, Figure 1. Both teams will work closely together throughout the duration of the project, but maintain different organizational structures and chains of command. The role of the implementation and evaluation team are specified above in Section 3.1.1, “Implementation Team” and Section 3.1.2, “Evaluation Team.”

**3.2.1 Implementation Team Role Specification**

***Project director:*** The project director is the person who is responsible for the overall implementation of the project and he reports to the UHMG Managing Director through the Director Technical services. The project director is responsible for ensuring that there is a work plan for implementation and this work plan is implemented by the relevant coordinators and officers. He is responsible for ensuring proper financial management by reviewing and authorizing expenditures related to the project, ensuring all advances are accounted for and UHMG, 3i.e and Harvard financial guidelines are followed. He is responsible for ensuring reports are prepared and submitted in accordance with the grant agreement. He supports and supervises the study coordinator, FSW coordinator and project officers.

***The study coordinator:*** The study coordinator is responsible for supporting the research and data related components of the project. He provides support in the identification of the study area, study participants and working with the International Research Consortium in the sampling component of the study. He is responsible for supporting the research team in collecting and compiling the baseline data, monthly client related data on HIV testing and the impact assessments. He is responsible for ensuring the relevant data collection tools are in place.

***The FSW coordinator:*** She is responsible for the coordination of all FSW related components of the study. This includes working with the FSW organization in mapping the hot spots, management of peer educators and following up study participants. She is responsible for organizing regular meetings with the FSW organizations and peer educators. The coordinator will also support training, mentorship and support supervision of the peer educators.

***The project officer:*** She is responsible for ensuring that the intervention and control groups received the standard of care package. She will support a team of 6 counselors to offer HIV counseling and referral services to FSWs in the participating hot spots. She will coordinate data management at the hot spots; compile weekly and monthly reports on project implementation. She will alert the project director on any emerging issues that need to be managed.

***Counselors:*** The counselors will support peer educators in the recruitment of potential study participants, support the research team in selection of study participants and in the collection of baseline information. The counselors will avail 24 hour telephone counseling services to the study participants and implement the standard of care at the selected hot spots.

**3.2.2 Evaluation Team Role Specification**

***Principal Investigator:*** The Principal Investigator for this study is based at the Harvard T.H. Chan School of Public Health; he has worked as an epidemiologist and health economist in Sub-Saharan Africa for more than 10 years. He has an extensive history of successful collaboration working with in-country implementation teams and providing rigorous evaluation of ongoing proposed projects using both experimental and quasi-experimental methods. For this project he is responsible for finalizing the design of the study and the desired analysis that will result from data collect through this study. He is also responsible for dealing with unlikely adverse events that might occur as a result of this study.

***Co-Principal Investigator:*** The Co-Principal Investigator is responsible for working closely with the Kampala-based Implementation team as data collection is ongoing to ensure the quality of the data collected. The co-PI will help train the research assistants for the study and will help oversee their activity in the field. He will add valuable feedback on the design of the study and help with analysis of the data and presentation of the results (in both oral presentations, policy briefs, and academic publications).

***Co-Investigators:*** The three Co-Investigators are primarily responsible for the design of the study protocol and survey instruments, training of research assistants, oversight of data quality, and the analysis of the collected data. They will work closely with the Implementation team and co-PI to ensure everyone understands the study design and the proposed analyses. They will report the results of the study in academic journals as well as international conferences in collaboration with the Ugandan colleagues.

***Research Assistant:*** The research assistant will help coordinate weekly meetings, edit study documents, help develop the online data platform, and help with data analysis as well as publication of study results.

**3.2.3 Oversight of Data Collection**

The study coordinator (Implementation team) will oversee all data collection activities. He will work with the FSW coordinator and project officer to collect information relevant to the project. He will develop and avail tools to be used by the peer educators and counselors to collect data. The tools include client cards, counselors’ client register and peer educators’ register. The counselors and peer educators will provide a monthly summary report on the number of FSWs. The project officer will collect HIV Counseling and Testing, HIV care and linkages data from the participating health facilities which is reported through the national Health Management Information System (HMIS). Figure 2 depicts the oversight of data collection for the HSPOT study


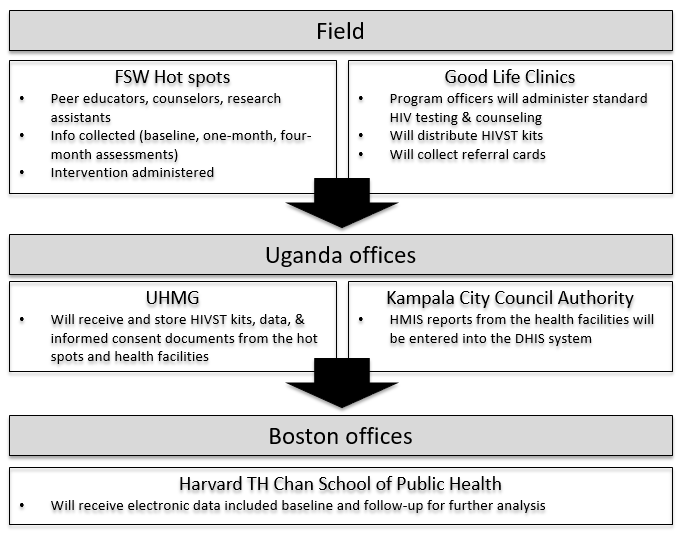


***Figure 2.*** *Oversight of data collection for the HSPOT study.*

**3.3 Study Setting**

The proposed study will take place in Kampala, the capital city of Uganda. In 2014 the population of Kampala was roughly 1.5 million people and in 2011 HIV prevalence was estimated to be around 9.5% in women and 4.1% in men. At the national level, prevalence of HIV among female sex workers (FSW) is estimated to be roughly five times greater than HIV prevalence among the general population; 35% compared to 7% respectively.

Kampala has a booming sex industry. Over the past four years, FSW outreach programs conducted by UHMG in Kampala has identified roughly 22,000 unique FSWs and nearly 180 hot spots – a number that continues to rapidly increase with time as individuals profit from these business-driven ventures. Hot spots are defined as places where FSWs and clients meet to conduct business – they include brothels, bars, hotels, lodges, and discotheques. Hot spots have clear structures and a lead individual who coordinates sex business.

This study will be conducted at FSW hot spots throughout Kampala’s five divisions, Figure 3. From these hot spots, and from pre-existing FSW peer educator programs, we will recruit and train FSW peer educators who will subsequently recruit other FSWs from these locations. We plan to talk with and engage the hot spot leader to gain permission before conducting research in their business area. At each hot spot we plan to rent a room or two (by the day) where both qualitative interviews and quantitative questionnaires can be conducted in private.

The study will also utilize Good Life Clinics to refer FSWs to for confirmatory testing, linkage to care and HIVST kit distribution (for the coupon study arm). Good Life Clinics are private health facilities affiliated with UHMG and funded by USAID to provide key populations friendly services. In Kampala there are 21 Good Life Clinics that all have prior experience working with key populations, especially FSWs. The Good Life Clinics differ from the public health facilities on the basis of ownership, most are sole proprietorships; are open 24 hours and provide a service with a profit and social good motive.


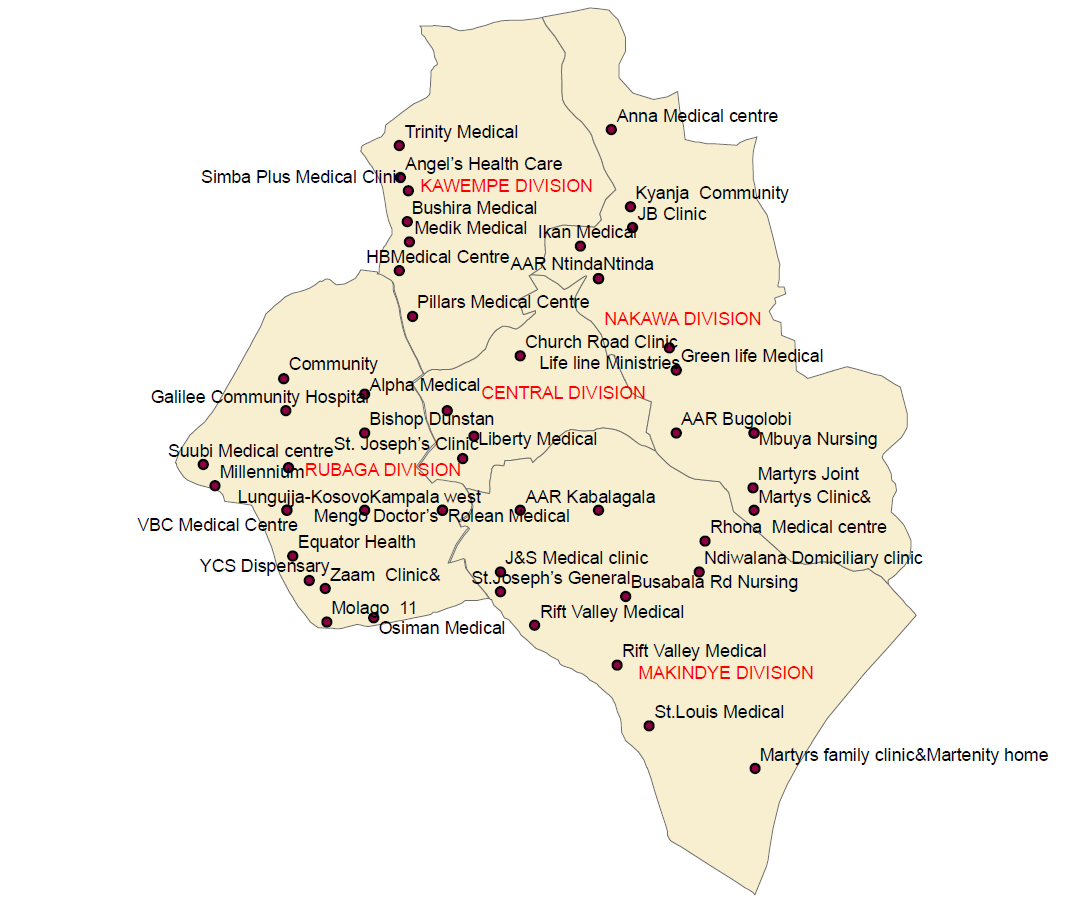


***Figure 3.*** *The five division of the Kampala City Authority: Rubaga, Kawempe, Nakawa, Makindye, and Central. Black dots show the distribution of Good Life Clinics within each division*

**4. PARTICIPANT FLOW**

- 1. **Study Timeline**

The target time period for completing the study is 15 months.

| **Date** | **Planned Activities** |
| --- | --- |
| January 2016 | - Meetings with key stakeholders, including policymakers and local government officials |
| April 2016 | - Finalization of the MOP, 1^st^ meeting of the scientific oversight committee - Submit Harvard & Uganda IRB |
| June 2016 | - HSPH IRB approval, Ugandan IRB approval |
| July-August 2016 | - Recruitment of peer educators - Final pre-study training of research assistants, peer educators, and study staff |
| September 2016 | - First enrollment |
| September 2016 to January 2016 | ENROLLMENT GOALS |
| Peer Educators | 120 (40 per arm) |
| Participants | 960 (320 per arm) |
| Qualitative Participants | 78 (26 per arm) |
| September 2016 | - Last enrollment |
| January 2016 | - Last 4-month follow-up visit |
| January 2017 to March 2017 | - Analyze, publish, and disseminate results - Results dissemination meeting with key stakeholders |

“Enrollment” is considered the date that the participant complete the informed consent procedure and baseline study activities. Note that a participant’s time on study begins to be counted at Intervention Visit 1 (Week 0; see Section 4.2).4.2

**4.2 Study Visit Schedule**

| ***Study Time Point*** | ***Visit By*** | ***Activities*** |
| --- | --- | --- |
| **Recruitment** | Peer Educator | - Peer educator discusses study with potentially eligible participant and refers them to study staff |
| **Enrollment** | Research Assistant | - Assessment of eligibility - Informed consent (if eligible) - Baseline quantitative survey - Baseline qualitative survey (~5%) |
| **Randomization** | Research Assistant | - Randomization of peer educator and their cohort of participants once all 5 group members are enrolled |
| **Intervention Visit 1**  **(Week 0)** | Peer Educator | - Group-based intervention - HIV prevention counseling and distribution of condoms - Training on HIV self-test use (in HIVST arms) - Distribution of HIV self-tests or coupons (in HIVST arms) |
| **Intervention Visit 2**  **(Week 2-3)** | Peer Educator | - Screening for adverse events - Discussion of any difficulty with HIVST kit use (in HIVST arms) - Referral to care and standard HIV testing - Distribution of condoms |
| **One Month Visit**  **(Week 4-5)** | Research Assistant | - One month quantitative survey - One month qualitative survey (~5%) - Screening for adverse events |
| **Intervention Visit 3**  **(Week 6-7)** | Peer Educator | - Screening for adverse events - Discussion of any difficulty with HIVST kit use (in HIVST arms) - Referral to care and standard HIV testing - Distribution of condoms |
| **Intervention Visit 4**  **(Week 10-12)** | Peer Educator | - Screening for adverse events - Discussion of any difficulty with HIVST kit use (in HIVST arms) - Referral to care and standard HIV testing - Distribution of condoms - Distribution of second HIVST kit or coupon (in HIVST arms) |
| **Four Month Visit**  **(Week 16-18)** | Research Assistant | - Four month quantitative survey - Four month qualitative survey (~5%) - Screening for adverse events - HIV status assessment |

- 1. **Eligibility Requirements**

*Inclusion Criteria (All must be met)*

- 18 years of age or older on the enrollment visit date
- Reports exchanging sex (vaginal, anal, and/or oral) for money or goods at least once in the past month
- Self-reported HIV negative status and no recent (<3 months) HIV testing OR self-reported HIV unknown status
- Member of the hot spot where recruited for at least one month AND plans on remaining in the area for the next 4 months
- Have never used an oral HIVST kit
- Willing to participate in peer education sessions on a monthly basis over the 4-month study period and to participate in study assessments
- Of sound mind and not under influence of drugs or coercion

*Exclusion Criteria (Any excludes)*

- Less than 18 years of age on the enrollment date
- Has not exchanged any from of sex in the past one month
- Self-reported to be living with HIV
- Self-reported HIV negative status and reports testing within the last 3 months
- Planning to move out of geographic area within 4 months
- Concurrently participating in another HIV prevention study
- Meets criteria but does not wish to participate
- Not willing or able to provide informed consent
- Other (to be documented)
  1. **Recruitment**

Recruitment will be performed by peer educators. Peer educators will be recruited from a variety of FSW hot spots equally spaced throughout Kampala’s five different divisions. During the training process, peer educators will be instructed on the eligibility criteria for the trial, and will be asked to identify 10-12 individuals from their social network to refer for enrollment into the study and their group. The peer educator will be asked to present key details of the study to the potential participant, and then will instruct them to contact the study staff for enrollment procedures (Appendix A.1; Recruitment Script). Peer educators can stop recruiting FSWs once eight of the recruited individuals have enrolled in the study. This study group will then be randomized to one of the three treatment arms. Peer educators will attempt to recruit the 8 eligible participants for their group within one-week period to minimize losses between recruitment and enrollment, but will be allowed up to two weeks if recruitment of eligible individuals is proving difficult. Formal enrollment will be done by the research assistant, as detailed below.

- 1. **Enrollment**

Enrollment is a multi-step process that is conducted by research assistants that begins when the recruited potential participant meets with the research assistant to assess their eligibility, formally enroll them in the study, and complete the baseline questionnaire (Appendices A.2 – A.5). It will be explained to participants that the enrollment and baseline assessment can take up to 2 hours, or possibly up to 3 hours if they are selected for inclusion in the qualitative in-depth interview baseline assessment, and that they will be compensated for their time.

- - 1. **Eligibility Assessment**

Eligibility will be assessed by the research assistant during the enrollment visit. The research assistant will review a checklist of inclusion and exclusion criteria with the participant to ensure that they meet each of the criteria (Appendix A.2; Eligibility Assessment). In particular, the research assistant will ensure that the participant is not already participating in the study by screening for dual enrollment. Participants will also be asked if they have ever participated in any HIV research before, and if they report that they have, they will be asked to report the nature of the project they were involved in, when it occurred, and where. This is an attempt to screen out individuals who are already participating in other HIV prevention studies.

- - 1. **Consent and Randomization**

The informed consent process will begin after ascertainment that all eligibility criteria have been met. The research assistant or study coordinator will explain the nature of the study to the participant, the primary research questions that are being asked, the randomization procedure, the content and frequency of intervention and study visits, and the potential risks and benefits of participating in the study. The participants will be assured that participation in the study is entirely voluntary, and that she can withdraw at any time if she feels uncomfortable without risking access to care or other services or any penalty. Furthermore, she will be told that she may elect not to answer any questions in surveys that she feels uncomfortable answering without ending her participation in the study. Written informed consent will then be obtained for each participant and contact information for consenting individuals will be obtained (Appendix A.3; Informed Consent and Appendix A.4; Participant Contact Form).

A random sub-sample of approximately 5% of individuals enrolled will be asked to enroll in to the qualitative sub-study (with a target enrollment of 15 participants per arm). If the participant has been randomly selected for the qualitative sub-study, the research assistant will be prompted by the tablet to enroll them into the sub-study. The research assistant will explain the nature of the sub-study to participants and explain that it will involve 2 in-depth interviews (at baseline and four months) and that each in-depth interview will take approximately 45 minutes to one hour to complete. Research assistants will explain to participants that participation in the sub-study is voluntary and they can elect to participate in the overall study but not the sub-study. If a participant chooses not to participate, another study participant will be randomly selected to participate in the qualitative sub-study in their place. Participants who enroll in the qualitative sub-study will be compensated additionally for their time in the sub-study.

Randomization to one of the three study arms occurs once all subjects have been enrolled in a peer educator’s cohort group, with a target enrollment of 8 participants per group. If a peer educator is unable to enroll 8 participants within a two-week timeframe, she will be allowed to proceed with a group of 7, although effort will be made to ensure that each peer educator meets her enrollment targets, including oversight of recruitment progress and financial incentives for meeting targets (intended to fairly compensate peer educators for the time that it takes to recruit). Due to the high mobility of FSWs, we are assuming that we will only be able to retain 75% of our study participants of the four-month duration of the study. Our study has been powered to detect meaningful effect sizes for clusters of 6 study participants per peer educator. Once the last participant has been enrolled in a peer educator’s group, the group will be assigned the next identification number from the randomization list, which will assign the group to one of the three study arms.

It is important to note that peer educators will be recruited from hotspots throughout Kampala’s five divisions. Specifically, of the 120 peer educators in our study, there will be 24 peer educators per Kampala division – each affiliated with a unique hotspot in that division. Then, when the peer educators and their enrolled study participants are randomized to a study arm, we will ensure equal representation of study arms by division – so in each division there will be 8 peer educators randomized to the control group, 8 peer educators randomized to the fixed distribution arm (e.g. “HIVST kit coupon”), and 8 peer educations randomized to the direct distribution arm (e.g. “HIVST kit direct”), Figure 4.


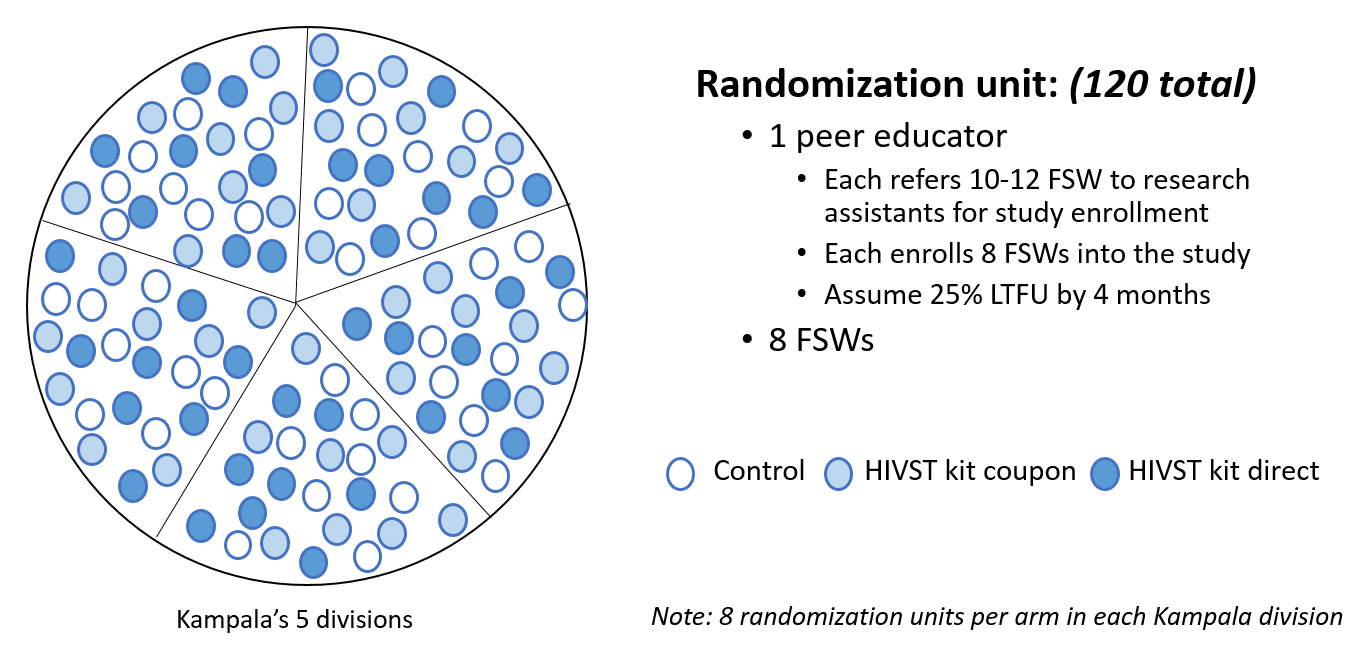


***Figure 4.*** Description of how the peer educator and FSW participants will be randomized to one of three study arms throughout Kampala’s five divisions.

- - 1. **Baseline Assessment**

Following assessment of eligibility and informed consent completion, the baseline assessment will be conducted with each participant. Every effort will be made to have the baseline assessment completed at the time of enrollment, but if a participant indicates that she wishes to return at a later date, she may do so as long as it is prior to Intervention Visit 1.

All participants will conduct the quantitative baseline questionnaire (Appendix A.5; Baseline Questionnaire). This assessment will be conducted via a tablet. The assessment will be completed in a face-to-face interview, with the research assistant conducting the interview and collecting the data directly on the tablet. The questionnaire has been tested and should take roughly 40 minutes to complete.

If the participant is randomly selected for participation in the qualitative sub-study, they will be asked to complete the in-depth interview on the enrollment/baseline assessment day. This interview will be conducted by the research assistant in a quiet, private space and will be audio recorded with the participant’s permission (part of the consent process, Appendix B.1; Baseline Qualitative Guide). The qualitative baseline interview should take approximately 45 minutes to an hour to complete.

- 1. **Follow-up Visits**

After enrollment and the baseline assessment, follow-up contact with the study is broadly divided into visits conducted by peer educators (Intervention Visits) and research assistants (One-month and Four-month Visits).

- - 1. **Intervention Visits**

Intervention visits will be conducted by the peer educator and will occur 4 times over the course of the study. The first intervention visit is expected to last approximately 45 minutes to one hour to accommodate training on HIV self-testing for those in the self-testing arm, whereas the remainder of the intervention visits are expected to last approximately 30 minutes. The first intervention session will be conducted in a group format, with all participants in a peer educator group attending the visit at the same time. They will be scheduled by peer educators with the help of the research assistant if necessary.

Intervention visits following the initial session will be conducted privately with participants, as they will include screening for adverse events including violence. The peer educator will schedule a follow-up intervention visit at two weeks after the first intervention session, then once monthly over the next two months. All intervention visits will be documented and recorded (Appendix A.6; Peer Educator Intervention Form). Participants will receive condoms as part of the peer counseling session, but they will not be compensated for attending these visits.

Please see more details on the intervention visits under Section 6, “Intervention Components.”

- - 1. **One-month and Four-month Visits**

Participants will complete two follow-up visits, at one-month after the first intervention session and at four-months after the first intervention session with a research assistant. Participants will be allowed a window of two weeks before or after the one or four-month date to complete the assessment. Participants will be reminded of their appointments through phone calls and their peer educators, who will work to ensure they complete their assessment visits on schedule. If a participant fails to return for one of the scheduled visits, they will be able to reschedule and the peer educator will counsel them that it is important to return for additional assessment visits. At each of these visits research assistants will conduct a one-month and four-month questionnaires, which should take approximately 30 minutes and 40 minutes respectively (Appendix A.7; One-month Questionnaire and Appendix A.8; Four-month Questionnaire). Participants will receive compensation for each of these questionnaires the complete with the research assistant.

If the participant was randomly selected for participation in the qualitative sub-study, they will be asked to complete another in-depth interview at the four-month visit. This interview will again be conducted by the research assistant in a quiet, private space and will be audio recorded with the participant’s permission (Appendix B.2; Four-month Qualitative Guide). The four-month baseline interview should take approximately 45 minutes to an hour to complete.

- 1. **Adverse Events and Dropout**
     1. **Adverse Events**

Adverse events will be monitored throughout the course of the project via several mechanisms. During the first intervention session and during the informed consent procedure, participants will be informed of possible adverse events that could occur. First session counseling will include strategies for dealing with violence, and it will be emphasized that participants should contact their peer educator or a member of study staff if any adverse events occur to them during the course of the study.

In the case of any adverse event, it will be reported by the peer educator to the research assistant, who will fill out an adverse event reporting form (Appendix A.9; Adverse Event Reporting Form), and adverse event narrative form if appropriate (Appendix A.10; Adverse Event Narrative Form). Adverse events can also be directly reported by study participants via a HSPOT hotline, which will be shared with the study participant at enrollment (Appendix A.16; Template HIV Counselor Hotline Card), can be called toll free, and will be monitored by trained HIV counselors throughout the duration of the study. Any adverse event requiring a narrative form will be reported to the principal investigators within 24 hours.

- - 1. **Participant Death**

Any patient death that occurs during the study period will be reported by the study coordinator to the Ugandan and Harvard principal investigators and the scientific oversight committee within 24 hours. If appropriate, the study arm of the participant will be disclosed to scientific oversight committee, with a copy of the serious adverse event narrative form (Appendix A.10; Adverse Event Narrative Form).

- - 1. **Patient Dropout**

The criteria for dropping out of the study include the participant declaring that they are no longer interested in participating in the study or if they move out of the study area. Missing a study visit does NOT mean that the participant has dropped out of the study. An adverse event, with the exception of participant death, also does not mean that the participant has dropped out of the study. In the case of participant dropout, the Participant Dropout Form (Appendix Form A.11; Patient Dropout Form) should be filled out.

1. **HIV SELF-TESTING KITS**

HIV self-testing kits will be acquired from OraSure, which produces OraQuick. The OraQuick oral HIV self-test is an FDA-approved oral swab in-home test for HIV-1 and HIV-2. The HIV self-test kits are completely self-administered. They involve collection of oral fluid from the gums with the swab provided in the kit, placing the tube into a vial with reagent, and waiting 20 minutes to read the result. The test kit contains a negative control; therefore, a negative test will show one line on self-test stick. A positive result will be indicated by the presence of two lines.

During the intervention, peer educators will provide basic verbal training on the use of the HIV self-test kits, however they will not provide extensive training so as to mimic real-life scenarios where extensive training on the use of the self-test kits is generally unavailable.

Pictorial instructions translated into local languages will be provided to participants. These instructions will be in the test kits themselves. Therefore, in the fixed distribution point arm, participants will not receive the instruction sheet unless they pick up a test kit from the fixed distribution point. The instructions will cover both steps for accurate use of the HIV self-test as well as instructions for accurately interpreting results, and what to do with either a negative or a positive HIV self-test. These instructions can be found in Appendix G.1; OraQuick Oral HIVST kit instructions.

Test kits will be procured through the OraSure Company and supplied by study staff. All testing kits will be stored at the UHMG warehouse in Kampala. In the fixed distribution arm, study staff will meet with participating Good Life Clinics prior to the initiation of the study. Staff at the Good Life Clinics will be trained on the study procedures and HIV self-testing during a half-day training session. UHMG Good Life Clinics have already been sensitized to working with key populations, especially FSWs, through previous USAID funded projects. This sensitization will help to protect the confidentiality of study participants.

1. **INTERVENTION COMPONENTS**

**6.1 Intervention Visit One**

Intervention Visit One will be a group-based intervention. The peer educator will meet with all participants in her group at the same time, and will review the study with the participants, and then will provide a brief peer education session related to HIV prevention and will distribute condoms to all participants. In the HIV self-testing fixed distribution and direct distribution arms, the peer educator will then provide a brief overview of HIV self-testing, will show participants the kits, and then will provide them with either the kit or the coupon for collecting the kit. All distributed HIV self-test kits will come with an unlabeled, opaque (e.g. black) sealable bag that study participants can use to dispose of used HIV self-test kits. These bags will be provided and explained to study participants by the peer educators. Since used HIVST kits are not considered medical waste (there is no risk of HIV infection to others if found), they can be disposed of in the municipal waste.

In all arms, the peer educator will discuss with participants where to get HIV testing services, and will discuss strategies for accessing services should participants have any hesitations about accessing the existing HIV testing services. Intervention Visit One is expected to last 30-45 minutes.

**6.2 Intervention Visits Two and Three**

Intervention Visits Two and Three are brief, one-on-one check-in visits that are conducted by the peer educator. The purpose of these visits is to check in with participants to see if they have any questions about the HIV self-test or the study and to screen for adverse events. Peer educators will distribute condoms in all arms and will complete a brief adverse event screen with each participant. Intervention Visits One and Two are expected to last approximately 15 minutes.

**6.3 Intervention Visit Four**

Intervention Visit Four is a one-on-one visit with the peer educator, and is the final Intervention visit. During this visit, the peer educator will screen for adverse events and will discuss any questions the participant has related to the study. She will then offer the participant a second HIV self-test kit or coupon in the direct distribution and fixed distribution arms, respectively. In all arms, the peer educator will provide condoms. Intervention Visit Four is expected to last approximately 15 minutes.

A guide for the various peer educator intervention visits is included in Appendices F.1 – F.3; Peer Educator Intervention Visit Guides.

1. **KEY INFORMANT FOCUS GROUPS**

***Peer educators will participate in focus group discussions (FGDs) prior to initiating training on study procedures as well as completion of the study.***

Key informants (peer educators) will complete a qualitative focus groups prior to initiating training on study procedures as well as at the completion of the study. The goal of the key informant focus groups is to elicit community norms on HIV testing and understand barriers and facilitators to HIV testing. At the end of the study, the goal of the key informant focus groups will be to develop an understanding of what was acceptable related to the intervention and what was not, and to elicit if community norms changed as a result of the intervention. Peer educators will be asked to participate in the focus groups before the study begins and willing participants will go through the informed consent process (Appendix C.1; Key Informant Informed Consent). The same consenting individuals will be asked to participate in focus groups upon study completion. Key informant focus group guides can be found in Appendix C.2; Pre-Study Key Informant Focus Group Guides and Appendix C.3; Post-Study Key Informant Focus Group Guides.

1. **ACCURACY STUDY**

During peer educator training, willing peer educators will undergo an assessment to understand the accuracy of HIV self-testing kits in this population. Although previous work has shown high sensitivity and specificity of the HIV self-test, the accuracy with which participants go through the steps of completing the tests after a brief training is unknown. We therefore propose to conduct an accuracy study with peer educators that is incorporated into the training we plan to conduct with them. On the first day of training, peer educators will receive basic information about HIV self-testing, will see a demonstration of the HIV self-test kit, and will receive written and pictorial instructions for their use. They will be informed that on the next day there will be an opportunity to participate in a study, in which they will test themselves for HIV using the HIV self-test kit while a study staff member observes them doing so. Participants will be informed that the study will take place in the morning, before the second day of training. Participants will be compensated for the additional 1 hour that it is anticipated to take to participate in this study. If they do not wish to participate in this study, they will be free to leave following completing the training.

A brief overview of the study will be presented to participants on the morning second day, and those who elect to participate will go through an informed consent process with the research assistant (Appendix D.1; Accuracy Study Invormed Consent). Following this, the participant will be asked to perform the HIV self-test in a private room, with the research assistant. The research assistant will observe the participant going through the steps of HIV self-testing by following a standardized checklist (Appendix D.2; Accuracy Study Assessment). The participant will have training materials available to her, such as the instructions for test kit use (Appendix G.1; OraQuick Oral HIVST kit instructions), but will be instructed to not to ask the research assistant questions about use of the test until the end. Once all steps of the test are complete, participants will be asked to place the used test kit in a black bag and hand this to the research assistant. All used participant testing kits will be mixed together in a larger bag so it is not possible to distinguish with test kit was used by a particular study participant. After a 20-minute waiting period (the time it takes for test results to appear), these de-identified used oral HIV self-test kits will be randomly distributed back to peer educators participating in the accuracy assessment for evaluation. Participants will then be asked to fill in a simple form that involves circling the result of the test they were given (positive or negative), placing this form with the HIV self-test in a black bag, and handing this to the research assistant (Appendix D.3; Accuracy Study Results) .

Because there will be no information identifying the participant on either the form or the black bag, there will be no way for the peer educator or study staff who read the results of the HIV self-tests to link the test result to a particular study participant, thus ensuring unwanted HIV status disclose does not occur and confidentiality of study participants is retained. Since we do not exclude HIV-positive peer educators in this study, we expect HIV prevalence in our peer educator population to match that of the national level for FSWs – around 35%. This ensures that we anticipate a critical number of HIV-positive test results to help with the education test kit interpretation, convince peer educators that oral testing can detect the HIV virus (compared to blood-based testing), and maintain the anonymity of HIV-positive peer educators. Also, having the study participant read anonymous HIVST kit result might reduce interpretation bias; for example, if they were already aware of their own HIV status, they might be inclined to report that.

All peer educators, regardless if they participate in the accuracy study, will be given the opportunity to test for HIV with standard services at the end of the training on day two if they are interested in knowing their HIV status.

1. **OUTCOME ASSESSMENT**

| **Outcome** | **Operationalization** |
| --- | --- |
| *Primary Endpoints* | |
| Use of HIV self-test | - Measured by buying back unused HIV self-tests after all participants have had the tests for at least one month |
| Tested for HIV in the past month | - Use of HIV self-test measured by buying back unused test tests after a one-month “wash out” period following the intervention to ensure that participant use of HIV self-test tests is not influenced by study design - Recent HIV testing measured by asking participants when they last tested and where (in both arms of the study) |
| Awareness of HIV status | - Measured using a three-step approach: 1) asking participants if they know what their status is2) offering participants a small financial gift if they can correctly tell the interviewer what their HIV status is; 3) confirming HIV status with a rapid test. If the status was correct, the participant receives the small financial gift. All participants will receive pre- and post-rapid test counseling |
| Empowerment/self-efficacy | - Quantities and qualitative questions - General Self-Efficacy Scale (GSE) - WONETHA enrollment at 4-months (voluntary) |
| *Secondary Endpoints* | |
| Linkage to care and confirmatory testing | - Measured in two ways: 1) during follow-up visits, asking participants if they received confirmatory testing and linked to care and 2) collection of referral cards linking individual HIV self-tests to individuals via a unique identification number |
| Sexual behaviors | - Measured via computer-assisted personal interview (CAPI), including number of sexual behaviors, event-level sexual behavior data, and condom use with primary and casual partners - Qualitatively assessed to understand sexual behaviors in relation to HIV testing, particularly within couples |
| HIV testing norms and stigma related to HIV testing | - Qualitatively assessed to understanding perceptions of HIV related stigma, barriers and facilitators to HIV testing, normative beliefs related to HIV testing |
| Cost-effectiveness | - Costing data for project implementation will be collected throughout the course of the intervention |
| *Safety Endpoints* | |
| Misuse of HIV self-tests | - Including difficulty conducting the test (i.e., mistakes in taking the test, incorrect use of components of the test), difficulty reading the test - Identified through interview and ongoing consultation with peer educators |
| Intimate partner violence | - Measured through surveillance and interviews by VHT members - Any intimate partner violence (including verbal, physical, or sexual) will be documented and reported |
| Difficulty using HIV self-tests | - Qualitatively explored during follow-up in depth interviews with participants - Recorded in an ongoing basis by peer educators who assist participants with using or interpreting tests |

*Primary endpoint*

Recent HIV testing will be ascertained by asking participants if they have tested for HIV in the previous month, regardless of where they tested, as well as where they tested for HIV. Recent HIV testing will be measured in all study arms. Testing in the last month is an important outcome, because the ultimate goal of this intervention is to achieve HIV testing increases. It is possible that HIV self-testing is crowding out other forms of HIV testing (i.e., the people who use an HIV self-test are those who would have tested via other methods had they counterfactually not received the HIV self-test). However, it is also possible that the HIV self-test intervention causally increases the uptake of HIV testing, either via HIV self-testing or other HIV testing methods. The evaluation aims to assess the total effect of the HIV self-testing intervention on HIV testing coverage.

*Secondary endpoints*

Use of HIV self-tests will be measured among individuals in the intervention arms of the study, and will be measured at Month 4. Accurate reporting of use of HIV self-tests may be affected by social desirability bias, and desire to report that the HIV self-tests were used as intended. We propose an innovative buy-back program in which research assistants will visit the homes of individuals who did not use the HIV self-test after one month from the original visit. During this visit, a research assistant will ask participants whether or not they used the HIV self-test. The research assistant will then offer the participant an incentive to “buy back” any unused HIV self-tests. The purpose of this incentive is to encourage individuals to return unused tests, thus allowing for an unbiased estimate of actual use of HIV self-tests. To avoid unintended consequences or spillovers that could arise if participants hear from other participants that researchers are buying back unused tests, follow-up of the intervention will not occur until one month after the last participant is enrolled. This buy back offer is not expected to bias the results of the study because of two study design features. First, we will not announce to participants that tests will be bought back, so they will not be aware that this will be an option. Second, we will not start outcome assessment until one month after the last woman is enrolled. We anticipate that the majority of individuals who will use the HIV self-test will use it within the first month of receiving it.

In addition to measuring use at all of HIV self-tests, we will record any issues that arise during the intervention period with the HIV self-test itself. Peer educators will be trained on the use of the HIV self-tests, and participants will be informed that if they have any issues with the use or interpretation of results of the test, they can contact their peer educator for help. All problems arising with use of the tests, including inaccurate usage and difficulty with interpretation of results, will be documented. At the follow-up visit, research assistants will ask participants if they had any trouble with the use of the HIV self-tests.

Knowledge of HIV status will be measured among individuals in both the intervention and control arms, and will be measured at Month 4. Self-reported HIV status is typically measured with a large degree of error. Factors including both social desirability bias and lack of awareness of status may bias self-reported estimates of HIV status. Previous studies have shown the positive predictive value of reporting a positive HIV status is quite high, indicating that people who say they are HIV positive typically are, but that the negative predictive value is considerably lower, suggesting that there is a significant subset of the population that is positive but self-reports being negative. This could be either due to hesitancy to disclose true status, or lack of awareness of status. To correctly measure knowledge of HIV status, it is necessary to tease these two reasons for incorrectly reporting HIV status apart. To achieve this, we propose a three-step method for ascertaining awareness of HIV status and then ascertaining whether a respondent’s perception of their own HIV status is correct. First, participants will be asked by research assistants if they know their status (but will not be asked what it is). Second, participants will be asked to report to research staff what their HIV status is. In order to incentivize participants to report their HIV status correctly, participants will be invited to play a game. If participants report an HIV status that matched the results of a rapid test, they will receive a small incentive. If the reported HIV status is not correct, they will not receive the incentive. The third component consists of a rapid test administered by the research assistant, to confirm HIV status. This incentive structure is not dissimilar to other HIV prevention incentives, and does not include disclosure of HIV status to anyone except the research assistant, who would learn HIV status if only the rapid test was performed.

Empowerment will be measured both quantitatively and qualitatively using various scales and questions, and then at the end of the four-month questionnaire, study participants will be given the opportunity to enroll in WONETHA and have the first year of membership waived. WONETHA (Women’s Network for Human Rights Advocacy) is a FSW owned and operated Kampala-based NGO, which has created a platform for FSWs in the area to organize themselves and improve their livelihoods. For an annual membership fee of UGX 10,000 (~$3 USD), FSW have access to three major programs provided by the organization: (1) a health outreach and support program, (2) an advocacy, partnership, and knowledge management program, and (3) an economic empowerment and capacity development program. These programs are designed to give FSWs the opportunity to develop advocacy skills, understand their reproductive rights, build skills in entrepreneurship, and improve their literacy. We will use enrollment in WONETHA as a proxy for empowerment and can compare WONETA enrollment with the other quantitative and qualitative data we plan to capture on this theme. Three months after study completion we plan to follow-up with WONETHA to see if any of the women who became WONETHA members actually accessed any of the WONTHA services.

Linkage to confirmatory testing and care will be measured the follow-up visit for all individuals in both arms of the trial. Linkage to care will also be continuously assessed over the study period by peer educators, who will be trained and involved in facilitating linkage to care for FSW. At baseline and Month 1 peer education counseling sessions, peer educators will encourage FSW to go for confirmatory HIV testing, particularly if they have a positive result, and they will be encouraged to seek the help of the peer educator in accessing confirmatory HIV testing. The study team will work closely with participating Good Life Clinics in Kampala to train staff on provision of healthcare in a non-discriminatory and stigma-free way (which luckily, have already been trained on how to deal with key populations through other USAID funded projects). Peer educators will inform FSW that these healthcare centers are offering friendly care services, and will link them to confirmatory testing and care at these centers.

Every time a peer educator links a participant to care, either for confirmatory testing or HIV-related care, they will document that the participant was linked to care (Appendix A.12; Peer Educator Referral Card). In addition, during the final follow-up visit, research assistants will ask participants if they sought confirmatory testing or other care services after receiving the results of their home test. Finally, a referral card will be included with each HIV self-test that contains a unique identification number as well as contact information for the clinics participating in the study (Appendix A.13: Study Participant Referral Card). Participants will be instructed to give the card to the clinic staff, who will return the card to study staff to record the participant’s visit at the clinic.

Sexual behaviors will be measured at baseline and follow-up for all participants using quantitative methods, and for a random sample using qualitative methods. A sexual behaviors assessment battery will be completed by participants, and will include quantitative questions related to the number of sexual partners in the last 3 months and condom use with sexual partners in the previous months. A set of questions specifically related to sexual behaviors with the participant’s main partner and sex work clients will also be included, and will include condom use with that partner and concerns about HIV transmission with the partner. Given that computer-assisted self-interview (CASI) is thought to produce more valid responses than interviewer-administered surveys, the sexual behaviors assessment battery will be administered via tablet computer.

HIV testing norms and stigma related to HIV testing will be assessed qualitatively during in-depth individual interviews at both baseline and Month 4. These interviews will focus on themes including 1) normative beliefs related to HIV testing behaviors; 2) social and community stigma related to HIV; 3) barriers and facilitators to HIV testing; 4) personal experience with the HIV self-tests, including reasons for using or not using the tests; 5) disclosure of HIV status to intimate partners and sexual behaviors in relation to HIV status; 6) HIV testing self-efficacy, including self-testing self efficacy; and 7) suggestions for improvement of the HIV self-testing program. Data will be analyzed in Dedoose using a descriptive qualitative approach to characterize and describe the data at their natural level. Qualitative interview guides are found in Appendix B.1 (Baseline Qualitative Guide) and Appendix B.2 (Four-month Qualitative Guide).

Cost effectiveness will include collection of costing data throughout the intervention. Costs will be measured prospectively following an activity-based approach to costing. We will record the start-up costs of the intervention (such as supply chains for HIV self-tests, training of peer educators and clinics participating in the study) as well as running costs specific to the program (excluding evaluation-specific costs). Cost effectiveness will be analyzed in terms of cost per person recently tested for HIV, cost per person aware of their HIV status, and cost per person successfully linked to care following a positive test. To establish economic spillover effects of the intervention, we will collect information on health care expenditures, employment, income, and wealth during follow-up surveys administered by the research assistants. A template for collection of costing data can be found in Appendix E.1; Costing Data Template.

Adverse events and safety endpoints will be carefully monitored and documented throughout the study. We expect adverse events to be rare, and could include inaccurate use of the tests, accidental harm during specimen collection, or psychological harm as a result of the use of the test. Psychological harm may occur if individuals receive a positive HIV self-test and were not prepared for the results. Particularly given that counseling is not automatically included with HIV self-testing (as the benefit of HIV self-testing is that it can occur in the privacy of one’s own home), it is possible that some people may have reactions to a positive test, such as depression, anxiety, or thoughts of suicide. We will address this by having a 24 hour hotline that study participants will be given information about and encouraged to call at any point. This hotline will be monitored by trained HIV counselors that can address any particular questions the study participant may have and help the participant link to care (Appendix A.15: Template HIV Counselor Hotline Card).

The primary safety endpoints of concern results from the intervention are the possibility of psychological harm, intimate partner violence, or human rights violations. While UNAIDS has recently indicated there has been no evidence of any serious adverse events related to HIV self-testing (i.e., self harm, violence, or human rights violations), we will ensure a careful monitoring plan is in place to identify any serious adverse events that arise during the study immediately. Other adverse events could include coerced testing, unintentional or unauthorized disclosure of HIV status, or incorrect use of the HIV self-test. Intimate partner violence could arise if partners perceive women to be HIV positive because they are using the tests, or if HIV status is disclosed as a part of the program. FSW may be particularly vulnerable to coercive testing or violence, and thus a specific emphasis on monitoring safety and adverse events will be placed on continuous monitoring and ensure a safety net is in place for all women enrolled in the study.

Peer educators will monitor their assigned FSW at each study visit for any adverse events, and will be trained in screening for depression, anxiety, suicidality, and intimate partner violence. Any indication of adverse events during these monitoring visits will trigger a visit by a research assistant, who will visit the FSW and document the adverse event on study forms (Appendix A.9; Adverse Event Reporting Form and Appendix A.10; Adverse Event Narrative Form). Research assistants will then report the adverse events to the Ugandan Project Director and Ugandan PI. Any and all safety events that occur during the study related to mental health, intimate partner violence, discrimination, human rights violations, and any other adverse events classified as serious, will be communicated immediately to the principal investigators, scientific oversight group, and 3ie (the funder).

1. **PROTECTION OF HUMAN SUBJECTS**

The study will be reviewed and approved by the Institutional Review Boards at Harvard T.H. Chan School of Public Health the Mildmay Uganda Institutional Review Board. Thereafter, the study will be registered with the Uganda National Council of Science and Technology in Kampala, Uganda.

- 1. **Potential risks to participants**

Reasonably foreseeable risks, discomforts, and inconveniences to participants involved in this study include:

*Accidental disclosure of sex worker status*. Because this study is specifically for female sex workers, it is possible that participation in the study itself could result in accidental disclosure that the participant is a sex worker. Although we expect this to be rare, as no study materials that participants take home will include that it is a sex worker-specific study, it could be possible to link the participant to the study and thus unintentionally disclose her profession as a sex worker. This could have legal, economic, or social consequences. The arrest of sex workers does happen in Uganda because sex work is illegal. Even though we are taking precautions to ensure the anonymity of our study participants, it is possible participation in this study could result in arrest of a study participant.

Although it is highly unlikely that disclosure of the identity of a participant would have economic consequences, it is possible if such a disclosure results in loss of non-sex work employment or loss of financial support from family. The duration of accidental disclosure of sex work employment at worst could have life-long consequences if it causes disturbances within social or family networks.

*Accidental disclosure of HIV serostatus.* As with accidental disclosure of sex worker status, it is possible that there could be accidental disclosure of HIV status associated with participation in this study. While participants will be HIV-uninfected or not know their status, it is possible they will test positive in this study. This could potentially be embarrassing for participants or could cause them to be ostracized from their communities. If a participant is ostracized from their community or discriminated against because of accidental disclosure of HIV status, this could cause psychological harm. This could also cause economic harm if potential clients learn about the HIV positive status of the participant and decide not to use her services. This could have long-term consequences depending on the nature of the disclosure, although we expect that with linkage to support services and counseling, the duration of this potential adverse event will be minimized. All peer educators will also be trained on the importance of maintaining FSW confidentiality if one of their peers chooses to disclose their HIV status for assistance with linkage to care.

*Psychosocial harm as a result of learning HIV status.* It is possible that participants will learn for the first time that they are living with HIV as part of participation in this study. This could cause psychological harm, as learning of a new diagnosis may cause psychosocial distress, depression, anxiety, or even thoughts of or actions related to suicide. Based on the existing HIV testing literature, the risk of completed suicide as a result of learning about a new HIV diagnosis is very low. However, it is much more likely that participants will feel depression and anxiety as a result of learning their HIV status. We have provided the 24 hour hotline with access to trained HIV counselors that study participants can call at any point to discuss psychological concerns. Research assistants and peer educators will also be trained to screen participants for signs of depression and out psychological symptoms during assessments and interventions.

*Intimate partner violence as a result of HIV self-test use or HIV infection.* Participants may be at risk of intimate partner violence if the fact that they are using an HIV self-test kit makes their partner perceive they are positive, participating in the study, or otherwise causes arguments with partners (including commercial or non-commercial). Although we anticipate that any intimate partner violence as a direct result of study participation will be rare, it is possible that this could cause physical harm to participants (assault and battery) and/or psychological harm (depression, anxiety, trauma).

*Psychosocial harm as a result of participating in study-related activities.* It is possible that participation in surveys and qualitative questionnaires could cause psychological harm. For example, participants will be asked questions related to traumatic childhood experiences (such as childhood sexual abuse or other abuse), that may trigger psychological harm and anxiety. Participants will be asked about intimate partner and other forms of violence, including harassment, abuse, and rape that may trigger post-traumatic stress disorder or anxiety.

*Misinterpretation of HIV self-test result.* It is possible that despite adequate training of peer educators, the peer educators might not correctly explain the use of the oral HIV self-test kit or FSWs might misinterpret the correct instructions. This could led to improper use of the test kit, incorrect test results, and consequently a misperception of ones HIV status. If a FSW is self-perceived HIV-negative when she is in fact positive, she is delaying necessary linkage to care and might have unprotected sex with a self-proclaimed HIV-negative client.

- 1. **Protection Against Risks**

To protect confidentiality, all data collected as part of this study will use a unique identification number, and a key linking the unique identification number to the participant’s identity will be kept separately from the database. The database will be secure and password-protected, and only authorized study staff will have access to it. The linkage key will kept in locked filing cabinets of locked UHMG offices in Kampala, will only be available to individuals with authorization, and will destroyed after data analysis and study completion. Research assistants will be able to enter data during interviews, but will not be able to go back and look at data that has been entered into the database. Data analysts (based at Harvard) will not have access to the key that links participant identification to the unique study identification number.

To protect associating participation in the study with being a sex worker, no materials developed for the study, including HIV self-testing kits, referral cards, pamphlets, or other study documents will include that this is a study for female sex workers. The informed consent document will be the only document that will state that this is a study for female sex workers, and this document will not be given to participants unless they ask for it. All other study documentation will state that this is a study looking at HIV testing in women.

Women experiencing adverse events, including depression, anxiety, suicidality, intimate partner violence, discrimination, or any other adverse event will be linked to counseling services and other care. We will work with WONTEHA, a key population advocacy agency based in Kampala, in the case of any arrests or legal action against participants to ensure they have proper legal counsel and services.

Adverse events associated with study participation will be monitored throughout the duration of the study by peer educators, research assistants, and other study staff. Any report of an adverse event will be immediately reported the Principal Investigator in Uganda and to the Principal Investigator at Harvard, as well as the Data and Safety Monitoring Board (as known as the SOC).

- 1. **Potential Benefits**

The primary direct benefit of the study is the potential to have access to an HIV self-test kit. For women who are in the intervention arms (fixed or direct distribution), they will be given up to two HIV self-test kits or coupons for collecting an HIV self-test kit. These kits are not otherwise currently available in Uganda. These participants will therefore have access to an HIV testing modality that is unavailable to the general public. Women participating in this study may learn their HIV status through this study, and linkage to care for those who test positive may be facilitated by participation. Without participating in the study, these women would not necessarily have access to a group that could assist them with linkage to care and confirmatory testing, and they may not have otherwise tested and thus may not have learned their status, allowing them to link to care earlier than they otherwise would have. Finally, participants in this study will have access to a social support network via the peer educators that they would not have access to outside of the study. This additional social support may have psychosocial or other benefits as a result of participating in the study.

The results of this study are expecting to inform national policy relating to oral HIV self-testing for female sex workers. As a priority population for HIV prevention in Uganda, the results of this study may have broad-reaching implications in terms of making HIV self-testing available to the population in general. If HIV self-testing is found to be efficacious and feasible as an alternative HIV testing strategy, this research may pave the way for national scale-up of HIV self-testing interventions. Conversely, if not found to be efficacious, resources that may have been spent on HIV self-testing will be redirected to other HIV prevention activities for female sex workers in Uganda. Thus, the benefits of the knowledge to be gained have primarily to do with policy generation for future HIV self-testing scale-up for this population.

1. **DATA MANAGEMENT**

Data will be stored only with a unique identification number that links participant data to their identity. All data will be completely de-identified, as the key linking unique study identification numbers to participant identities will be stored separately from the database. This key will be kept in locked filing cabinets of locked UHMG offices in Kampala, and will only be available to individuals with authorization (research assistants, the study coordinators, the Uganda project director, and the Ugandan principal investigator). No one with access to the de-identified dataset will also have access to the key, and the key will be destroyed upon completion of the study.

All data uploaded to the CommCare cloud server in-country will be encrypted and password-protected in accordance to the Level 4 data security and storage regulations. The de-identified data will then be uploaded to an encrypted password-protected FTP site on a daily or weekly basis and will be circulated to the Harvard HSPOT evaluation team for analysis purposes for the duration of the study. After the study ends, only de-identified datasets will remain available for analysis purposes after the end of the study.

Every effort will be made to be sure that participation in this study, and all records about participation, will remain confidentia.. Data will be collected by trained research assistants and fully de-identified as soon as possible. We will work with Dimagi’s CommCare to set up a data management system that meets the following requirements:

Raw electronic survey data will be immediately transferred once it has been collected on the Android-based tablets using a secure data transfer to the CommCare secure cloud server. Following the transfer, the data from the Android tablets will be automatically erased.

All identifying information will be separated from the raw electronic survey data immediately after collection and secure transfer to the cloud server, and a unique ID number will be assigned to each case. Coded, de-identified data files will be stored separately from the code list and identified data files to maintain confidentiality. Only the Uganda PI, will have access to the linkages to the underlying identifiable files. Identifiable electronic data will be encrypted, password-protected, and securely stored on the CommCare protected cloud server and one copy of the data will be stored on a password-protected computer, which will be designated as the Target Computer. De-identified data will be encrypted, password-protected, and securely stored separately from the identifiable data on the CommCare protected cloud server and on an encrypted password-protected FTP site.

All staff members of the study will be required to sign a data confidentiality agreement. The data will be stored in a relational database. Usernames and passwords are required to access the data. A security policy is used to ensure these passwords are updated on a regular basis.

Identifiable hard-copy data, including signed consent forms, will be stored in locked cabinets in access-limited rooms at the UHMG Kampala offices. All study data and computers used to analyze the data be password protected at all times. All electronic data, both on the CommCare secure cloud server and on any study computers, will be encrypted and password-protected. The information will only be accessible to the research team and will be available from a secure server upon completion of this process.

1. **STATISTICAL ANALYSIS**

As this is a randomized study, the primary analysis will be by intention-to-treat (ITT). The primary analysis for the quantitative data of the main study will involve a mixed-effects multilevel logistic regression model to account for clustering by peer educator group and study site. These models will include an indicator term for study arm (fixed distribution, direct distribution, or control). The outcomes (recent HIV testing and correct knowledge of status) will be modeled as dichotomous variables. Sensitivity analyses will be run to test for robustness of results to selection bias using Heckmann selection models using peer educator identity as a random effect, and adjustment for covariates if balance tests suggest an imbalance in baseline characteristics. All data will be analyzed in Stata version 14.0.

Qualitative data will be analyzed using a descriptive approach to characterize and describe data at their natural level. A codebook will be developed based on an iterative process by an initial evaluation and data immersion of transcribed and translated interviews. Codes will be structured with basic overarching themes. Data will be coded by at least two independent coders. Inter-coder agreement will be assessed via calculation of the kappa coefficient in Dedoose, an online platform for qualitative analysis. Data will be analyzed overall and by the following subgroups: 1) by study arm (for follow-up qualitative interviews); 2) by hotspot venue type (for baseline and follow-up); and 3) by nationality (Ugandan versus non-Ugandan native).

Accuracy study data will be analyzed using a descriptive approach. The number of participants accurately completing each step of the HIV self-test process will be calculated, and the total number who accurately use the HIV self-test will be reported. Finally, the data for participant interpretation of results versus study staff assessment of the result on the HIV self-test kit will be cross-tabulated and the sensitivity and specificity of the participant interpretation of the HIV self-test result versus the study member (gold standard) will be evaluated.

Costing analyses will estimate costs for each study participate based on the micro-costing data. Costs will be estimated from two perspective: that of the peer educator based HIV self-testing intervention (including peer educator salary, cost of testing kits, and informational materials), and a societal perspective (also includes costs incurred by study participants). The primary cost-effectiveness endpoint will be the incremental cost per individual who recently tested for HIV for the intervention arms compared to the control arm (clinic-based HIV testing). The incremental cost-effectiveness ratio (ICER) will be calculated as the difference in average cost per patient between the study arms (intervention minus standard-of-care), divided by the difference in the probability of testing for HIV between arms. Cost estimates in the intervention arm will include all resources required to achieve a particular end point, and cost for standard-of-care will be collected from local clinics that offer HIV testing and counseling. For all cost-effectiveness outcomes, measures of uncertainty will be achieved by bootstrapping the participant-level estimates of cost and effectiveness of end points (accounting for clustering). Results will be presented as confidence intervals and cost-effectiveness acceptability curves. Sensitivity analyses will be conducted to assess the robustness of the cost-effectiveness results to uncertainty in major costs and other determinants of intervention efficiency (e.g. wage levels, intervention uptake, etc.).

All data, including qualitative and quantitative data, will be completely de-identified prior to analysis, and the analytic datasets will not contain any identifiable or re-identifiable information.

**APPENDIX A: PARTICIPANT FORMS**

1. Participant Recruitment Script
2. Eligibility Assessment
3. Informed Consent
4. Participant Contact Form
5. Baseline Questionnaire
6. Peer Educator Intervention Form
7. One-month Questionnaire
8. Four-month Questionnaire
9. Adverse Event Reporting Form
10. Adverse Event Narrative Form
11. Patient Dropout Form
12. Peer Educator Referral Card
13. Study Participant Referral Card
14. Contact Information Card
15. HIVST Kit Coupon
16. Template HIV Counselor Hotline Card

**APPENDIX B: QUALITATIVE INTERVIEW GUIDES**

1. Baseline Qualitative Guide
2. Four-month Qualitative Guide

**APPENDIX C: KEY INFORMAITON FOCUS GROUPS**

1. Key Informant Informed Consent
2. Pre-Study Key Informant Focus Group Guide
3. Post-Study Key Informant Focus Group Guide

**APPENDIX D: ACCURACY STUDY**

1. Accuracy Study Informed Consent
2. Accuracy Study Assessments
3. Accuracy Study HIVST Kit Results Form

**APPENDIX E: CERTIFICATION AND STUDY MANAGEMENT FORMS**

1. Costing Data Template
2. Research assistant certification form
3. Peer educator certification form

**APPENDIX F: INTERVENTION VISIT GUIDE**

1. Peer Educator Intervention Visit Guide - Control
2. Peer Educator Intervention Visit Guide - Direct
3. Peer Educator Intervention Visit Guide - Fixed

**APPENDIX G: HIV SELF-TEST INSTRUCTIONS**

1. OraQuick Oral HIVST kit instructions
2. **REFERENCES**

1. Baral S, Beyrer C, Muessig K, *et al.* Burden of HIV among female sex workers in low-income and middle-income countries: a systematic review and meta-analysis. *The Lancet Infectious Diseases* 2012; **12**: 538–49. [↑](#endnote-ref-1)
2. Shannon K, Strathdee SA, Goldenberg SM, *et al.* Global epidemiology of HIV among female sex workers: Influence of structural determinants. *The Lancet* 2014; : 1–17. [↑](#endnote-ref-2)
3. Elmes J, Nhongo K, Ward H, *et al.* The Price of Sex: Condom Use and the Determinants of the Price of Sex Among Female Sex Workers in Eastern Zimbabwe. *J INFECT DIS* 2014; **210**: S569–78. [↑](#endnote-ref-3)
4. Morris CN, Morris SR, Ferguson AG. Sexual Behavior of Female Sex Workers and Access to Condoms in Kenya and Uganda on the trans-Africa Highway. *AIDS Behav* 2009; **13**: 860–5. [↑](#endnote-ref-4)
5. The Gap Report 2014. (UNAIDS, 2014). at http://www.unaids.org/sites/default/files/media_asset/UNAIDS_Gap_report_en.pdf [↑](#endnote-ref-5)
6. 6 UNAIDS country profile; Uganda. (2014). at http://www.unaids.org/en/regionscountries/countries/uganda [↑](#endnote-ref-6)
7. 7 HIV and AIDS Uganda Country Progress Report; 2013. (Uganda AIDS Comission, 2014). at http://www.uhasselt.be/Documents/UHasselt/onderwijs/internationaal/noord-zuid_2015/HIV_and_AIDS_Uganda_Country_Progress_Report_2013.pdf [↑](#endnote-ref-7)
8. 8 Donnell, D. et al. Heterosexual HIV-1 transmission after initiation of antiretroviral therapy: a prospective cohort analysis. The Lancet375, 2092–2098 (2010). [↑](#endnote-ref-8)
9. 9 Cohen, M. S. et al. Prevention of HIV-1 Infection with Early Antiretroviral Therapy. N. Engl. J. Med. 365,493–505 (2011). [↑](#endnote-ref-9)
10. 10 2014 Report on Costs of Treatment in the President’s Emergency Plan for AIDS Relief (PEPFAR). (PEPFAR, 2014). At <http://www.pepfar.gov/documents/organization/223163.pdf> [↑](#endnote-ref-10)
11. 11 Haber, N., Naidu, K., Pillay, D. & Barnighausen, T. HIV System Assessment with Longitudinal Treatment Cascade in KwaZulu-Natal, South Africa. (2014). [↑](#endnote-ref-11)
12. 12 Kamya, M. Cascade of HIV Care in Uganda. (2013). at <http://cfar.ucsf.edu/cfar?page=symposia-13-kamya-cascade&post=1> [↑](#endnote-ref-12)
13. 13 Fox, M. P. & Rosen, S. Patient retention in antiretroviral therapy programs up to three years on treatment in sub-Saharan Africa, 2007–2009: systematic review. Trop. Med. Int. Health 15, 1–15 (2010). [↑](#endnote-ref-13)
14. 14 Rosen, S. & Fox, M. P. Retention in HIV Care between Testing and Treatment in Sub-Saharan Africa: A Systematic Review. PLoS Med 8, e1001056 (2011). [↑](#endnote-ref-14)
15. 15 90–90–90 - An ambitious treatment target to help end the AIDS epidemic. At <http://www.unaids.org/en/resources/documents/2014/90-90-90> [↑](#endnote-ref-15)
16. 16 Uganda AIDS Indicator Survey 2011. (Ugandan Ministr of Health, ICF International, Center for Disease Control and Prevention, US Agency for International Development, WHO Uganda, 2012). [↑](#endnote-ref-16)
17. 17 HIV & AIDS in Uganda. at <http://www.avert.org/hiv-aids-uganda.htm> [↑](#endnote-ref-17)
18. 18 Summary of formative research in Kenya and Zambia. (3ie, 2015). [↑](#endnote-ref-18)
19. 19 Policy & Regulations for HIVST. HIVST.org at <http://www.hivst.org/policy-regulations-for-hivst-1/> [↑](#endnote-ref-19)
20. 20 Figueroa, C., Johnson, C., Verster, A. & Baggaley, R. Attitudes and Acceptability on HIV Self-testing Among Key Populations: A Literature Review. AIDS Behav.1–17 (2015). doi:10.1007/s10461-015-1097-8 [↑](#endnote-ref-20)
21. 21 Brown, A. N., Djimeu, E. W. & Cameron, D. B. A Review of the Evidence of Harm from Self-Tests. AIDS Behav.18,445–449 (2014). [↑](#endnote-ref-21)
22. 22 Peer education and HIV/AIDS: Concepts, uses and challenges. (UNAIDS, 1999). at <http://www.unaids.org/sites/default/files/media_asset/jc291-peereduc_en_0.pdf> [↑](#endnote-ref-22)
