# Supplementary material for: Direct provision versus facility collection of HIV self-tests among female sex workers in Uganda: A cluster-randomized controlled health systems trial
Source: PLoS Med. 2017 Nov 28;14(11):e1002458. doi: 10.1371/journal.pmed.1002458 (PMC5705079; doi:10.1371/journal.pmed.1002458)
Supplement: S1 Table — (DOCX) [file pmed.1002458.s003.docx]

**S1 Table. Loss to follow up and refusal to answer questions about HIV status at 1 month and at 4 months.**

| **Outcome** | **1 month, *n* (%)** | | | | **4 months, *n* (%)** | | | |
| --- | --- | --- | --- | --- | --- | --- | --- | --- |
|  | ***Direct provision,***  ***n* = 289** | ***Facility collection,***  ***n* = 321** | ***Standard of care,***  ***n* = 315** | ***p*-value**^1^ | ***Direct provision,***  ***n* = 262** | ***Facility collection,***  ***n* = 297** | ***Standard of care,***  ***n* = 301** | ***p*-value**^1^ |
| Loss to follow-up | 7  (2.4%) | 15  (4.5%) | 13  (4.0%) | 0.250^2^  0.870^3^  0.213^4^ | 34  (11.5%) | 39  (11.6%) | 27  (8.2%) | 0.307^2^  0.264^3^  0.964^4^ |
| Refused to answer questions about HIV status | 0 | 3  (0.9%) | 10  (3.2%) | ---  0.057^3^  --- | 3  (1.1%) | 9  (3.0%) | 8  (2.7%) | 0.987^2^  0.223^3^  0.244^4^ |

**Abbreviations:** *n*, total number of participants.

^1^Multilevel mixed effects generalized linear models (Poisson distribution, log link, robust standard errors), study arm fixed effect, peer educator random effect; intention-to-treat analyses.

^2^*p*-value for direct provision vs. standard-of-care arm.

^3^*p-*value for facility collection vs. standard-of-care arm.

^4^*p*-value for direct provision vs. facility collection arm.
