# Supplementary material for: Direct provision versus facility collection of HIV self-tests among female sex workers in Uganda: A cluster-randomized controlled health systems trial
Source: PLoS Med. 2017 Nov 28;14(11):e1002458. doi: 10.1371/journal.pmed.1002458 (PMC5705079; doi:10.1371/journal.pmed.1002458)
Supplement: S2 Table — RR, risk ratio. (DOCX) [file pmed.1002458.s004.docx]

**S2 Table. Effect size estimates: Risk ratios. RR, risk ratio.**

| **Outcome*^2^*** | |  | ***Direct provision vs.***  ***Standard-of-care*** | | ***Facility collection vs.***  ***Standard-of-care*** | | ***Direct provision vs.***  ***Facility collection*** | | **Joint significance test** |
| --- | --- | --- | --- | --- | --- | --- | --- | --- | --- |
|  |  | **Assessment** | **RR^1^ (95% CI)** | ***p*-value** | **RR^1^ (95% CI)** | ***p*-value** | **RR^1^ (95% CI)** | ***p*-value** | ***p-*value** |
| ***HIV testing*** | |  |  |  |  |  |  |  |  |
| Tested for HIV | | 1 month* | 1.33 (1.17-1.52) | <0.001 | 1.12 (0.96-1.32) | 0.148 | 1.18 (1.07-1.31) | 0.001 | <0.001 |
|  |  | 4 months* | 1.14 (1.07-1.22) | <0.001 | 1.11 (1.04-1.19) | 0.002 | 1.03 (1.01-1.05) | 0.015 | <0.001 |
|  | *Tested for HIV twice* | 4 months | 1.51 (1.29-1.77) | <0.001 | 1.24 (1.04-1.49) | 0.021 | 1.22 (1.08-1.37) | 0.001 | <0.001 |
| Used an HIV self-test | | 1 month | --- |  | --- |  | 1.21 (1.09-1.35) | 0.001 | <0.001 |
|  |  | 4 months | --- |  | --- |  | 1.05 (1.01-1.09) | 0.010 | <0.001 |
|  | *Used a self-test twice* | 4 months | --- |  | --- |  | 1.22 (1.06-1.40) | 0.005 | <0.001 |
| Tested for HIV at a facility^3^ | | 1 month | 0.14 (0.09-0.22) | <0.001 | 0.13 (0.08-0.21) | <0.001 | 1.07 (0.58-1.98) | 0.827 | <0.001 |
|  |  | 4 months | 0.25 (0.18-0.34) | <0.001 | 0.29 (0.23-0.37) | <0.001 | 0.85 (0.59-1.22) | 0.373 | <0.001 |
|  | *Tested for HIV at facility twice* | 4 months | 0.03 (0.01-0.09) | <0.001 | 0.07 (0.04-0.13) | <0.001 | 0.51 (0.17-1.53) | 0.227 | <0.001 |
| Tested HIV-positive | | 1 month | 1.05 (0.62-1.75) | 0.866 | 1.27 (0.74-2.19) | 0.386 | 0.82 (0.48-1.41) | 0.476 | 0.660 |
|  |  | 4 months | 0.95 (0.62-1.48) | 0.835 | 1.53 (1.00-2.36) | 0.050 | 0.62 (0.41-0.94) | 0.025 | 0.047 |
| ***Linkage to care^4^*** | |  |  |  |  |  |  |  |  |
| Sought medical care for HIV | | 1 month | 0.65 (0.30-1.41) | 0.275 | 0.50 (0.24-1.04) | 0.063 | 1.30 (0.54-3.15) | 0.557 | 0.557 |
|  |  | 4 months | 0.83 (0.49-1.41) | 0.482 | 1.01 (0.62-1.65) | 0.967 | 0.82 (0.46-1.44) | 0.488 | 0.488 |
| Initiated ART | | 1 month | 0.99 (0.37-2.67) | 0.991 | 0.76 (0.29-2.02) | 0.585 | 1.30 (0.46-3.73) | 0.619 | 0.619 |
|  |  | 4 months | 0.91 (0.46-1.81) | 0.879 | 1.15 (0.63-2.10) | 0.646 | 0.79 (0.41-1.54) | 0.490 | 0.490 |

*Pre-specified primary outcomes: any HIV testing at 1 month and at 4 months.

^1^Multilevel mixed effects generalized linear models (Poisson distribution, log link, robust standard errors), study arm fixed effect, peer educator random effect; intention-to-treat analyses.

^2^All testing and linkage to care outcomes self-reported since study start.

^3^Facility-based HIV testing included private and public healthcare facilities.

^4^For these outcomes, participants had to report both testing HIV positive and seeking HIV-related medical care or initiating ART. These outcomes were measured among all participants randomized, as defined by the intention-to-treat analysis.
