# Supplementary material for: Direct provision versus facility collection of HIV self-tests among female sex workers in Uganda: A cluster-randomized controlled health systems trial
Source: PLoS Med. 2017 Nov 28;14(11):e1002458. doi: 10.1371/journal.pmed.1002458 (PMC5705079; doi:10.1371/journal.pmed.1002458)
Supplement: S3 Table — PP, percentage point. (DOCX) [file pmed.1002458.s005.docx]

**S3 Table. Effect size estimates: risk differences. PP, percentage point.**

| **Outcome*^2^*** | |  | ***Direct provision vs.***  ***Standard-of-care*** | | ***Facility collection vs.***  ***Standard-of-care*** | | ***Direct provision vs.***  ***Facility collection*** | | **Joint**  **sig. test** |
| --- | --- | --- | --- | --- | --- | --- | --- | --- | --- |
|  |  | **Assessment** | **PP^1^ (95% CI)** | ***p*-val** | **PP^1^ (95% CI)** | ***p*-val** | **PP^1^ (95% CI)** | ***p*-val** | ***p-*val** |
| ***HIV testing*** | |  |  |  |  |  |  |  |  |
| Tested for HIV | | 1 month* | 24.2 (13.9 to 34.5) | <0.001 | 9.6 (-0.4 to 19.6) | 0.061 | 14.6 (4.4 to 24.9) | 0.005 | <0.001 |
|  |  | 4 months* | 12.9 (7.6 to 18.2) | <0.001 | 10.3 (5.2 to 15.4) | <0.001 | 2.6 (-2.7 to 7.8) | 0.342 | <0.001 |
|  | *Tested for HIV twice* | 4 months | 29.6 (19.1 to 40.2) | <0.001 | 14.5 (4.3 to 24.7) | 0.005 | 15.1 (4.6 to 25.7) | 0.005 | <0.001 |
| Used an HIV self-test | | 1 month | --- |  | --- |  | 16.1 (9.0 to 23.1) | <0.001 | <0.001 |
|  |  | 4 months | --- |  | --- |  | 4.2 (0.3 to 8.1) | 0.033 | <0.001 |
|  | *Used a self-test twice* | 4 months | --- |  | --- |  | 27.4 (10.0 to 44.8) | 0.008 | <0.001 |
| Tested for HIV at a facility^3^ | | 1 month | -56.8 (-66.3 to -47.3) | <0.001 | -57.4 (-66.6 to -48.2) | <0.001 | 0.6 (-8.9 to 10.0) | 0.909 | <0.001 |
|  |  | 4 months | -64.2 (-72.8 to -55.7) | <0.001 | -60.3 (-68.6 to -52.0) | <0.001 | -3.9 (-12.4 to 4.7) | 0.374 | <0.001 |
|  | *Tested for HIV at a facility twice* | 4 months | -42.1 (-50.9 to -33.3) | <0.001 | -40.8 (-49.4 to -32.3) | <0.001 | -1.2 (-10.1 to 7.6) | 0.781 | <0.001 |
| Tested HIV-positive | | 1 month | 0.4 (-7.5 to 8.4) | 0.915 | 4.0 (-3.7 to 11.7) | 0.310 | -3.6 (-11.5 to 4.3) | 0.374 | 0.539 |
|  |  | 4 months | -0.8 (-10.0 to 8.4) | 0.869 | 9.6 (0.7 to 18.6) | 0.035 | -10.4 (-19.6 to -1.2) | 0.027 | 0.043 |
| ***Linkage to care^4^*** | |  |  |  |  |  |  |  |  |
| South medical care for HIV | | 1 month | -2.6 (-7.4 to 2.3) | 0.307 | -4.1 (-8.8 to 0.7) | 0.091 | 1.6 (-3.3 to 6.4) | 0.528 | 0.234 |
|  |  | 4 months | -2.1 (-8.4 to 4.3) | 0.525 | 0.2 (-6.0 to 6.3) | 0.961 | -2.2 (-8.6 to 4.1) | 0.496 | 0.753 |
| Initiated ART | | 1 month | 0.2 (-3.8 to 4.2) | 0.908 | -0.9 (-4.8 to 3.0) | 0.653 | 1.1 (-2.8 to 5.1) | 0.577 | 0.838 |
|  |  | 4 months | -0.6 (-6.0 to 4.9) | 0.837 | 1.2 (-4.0 to 6.5) | 0.645 | -1.9 (-7.3 to 3.6) | 0.514 | 0.797 |

**Abbreviations:** CI, confidence interval; p-val, p-value; sig, significance.

*Pre-specified primary outcomes: any HIV testing at 1 month and at 4 months.

^1^Multilevel mixed effects generalized linear models, study arm fixed effect, peer educator random effect; intention-to-treat analyses.

^2^All testing and linkage to care outcomes self-reported since study start.

^3^Facility-based HIV testing included private and public healthcare facilities.

^4^For these outcomes, participants had to report both testing HIV positive and seeking HIV-related medical care or initiating ART. These outcomes were measured among all participants randomized, as defined by the intention-to-treat analysis.
