# Supplementary material for: Direct provision versus facility collection of HIV self-tests among female sex workers in Uganda: A cluster-randomized controlled health systems trial
Source: PLoS Med. 2017 Nov 28;14(11):e1002458. doi: 10.1371/journal.pmed.1002458 (PMC5705079; doi:10.1371/journal.pmed.1002458)
Supplement: S4 Table — RR, risk ratio. (DOCX) [file pmed.1002458.s006.docx]

**S4 Table. Sensitivity analysis: Pooled HIV self-testing arms versus the standard of care arm. RR, risk ratio.**

| **Outcome^2^** | | **Assessment** | ***Pooled HIV self-test arms*** | ***Standard-of-care*** | **RR^1^ (95% CI)** | ***p*-value** |
| --- | --- | --- | --- | --- | --- | --- |
| ***HIV testing*** | |  |  |  |  |  |
| Tested for HIV | | 1 month* | 533/610 (87.4%) | 226/316 (71.5%) | 1.22 (1.07-1.40) | 0.004 |
|  |  | 4 months* | 549/559 (98.2%) | 263/302 (87.1%) | 1.13 (1.05-1.21) | <0.001 |
|  | *Tested for HIV twice* | 4 months | 440/559 (71.3%) | 174/302 (57.6%) | 1.37 (1.16-1.60) | <0.001 |
| Tested for HIV at a facility^3^ | | 1 month | 55/610 (9.0%) | 211/316 (66.8%) | 0.13 (0.10-0.19) | <0.001 |
|  |  | 4 months | 131/559 (23.4%) | 259/302 (85.8%) | 0.27 (0.23-0.33) | <0.001 |
|  | *Tested for HIV at a facility twice* | 4 months | 13/559 (2.3%) | 136/302 (45.0%) | 0.05 (0.03-0.09) | <0.001 |
| Tested HIV-positive | | 1 month | 93/599 (15.5%) | 39/301 (13.0%) | 1.16 (0.73-1.84) | 0.520 |
|  |  | 4 months | 124/549 (22.6%) | 53/294 (18.0%) | 1.26 (0.85-1.86) | 0.244 |
| ***Linkage to care^4^*** | |  |  |  |  |  |
| Sought medical care for HIV | | 1 month | 30/559 (5.0%) | 25/301 (8.3%) | 0.57 (0.30-1.06) | 0.073 |
|  |  | 4 months | 64/549 (11.7%) | 37/294 (12.6%) | 0.92 (0.60-1.41) | 0.711 |
| Initiated ART | | 1 month | 23/599 (3.8%) | 13/301 (4.3%) | 0.87 (0.38-2.01) | 0.746 |
|  |  | 4 months | 46/549 (8.4%) | 24/294 (8.2%) | 1.04 (0.60-1.80) | 0.894 |

*Pre-specified primary outcomes: any HIV testing at 1 month and at 4 months.

^1^Multilevel mixed effects generalized linear models (Poisson distribution, log link, robust standard errors), study arm fixed effect, peer educator random effects; intention-to-treat analyses.

^2^All testing and linkage to care outcomes self-reported since study start.

^3^Facility-based HIV testing included private and public healthcare facilities.

^4^For these outcomes, participants had to report both testing HIV positive and seeking HIV-related medical care or initiating ART. These outcomes were measured among all participants randomized, as defined by the intention-to-treat analysis.
