# Supplementary material for: Direct provision versus facility collection of HIV self-tests among female sex workers in Uganda: A cluster-randomized controlled health systems trial
Source: PLoS Med. 2017 Nov 28;14(11):e1002458. doi: 10.1371/journal.pmed.1002458 (PMC5705079; doi:10.1371/journal.pmed.1002458)
Supplement: S5 Table — PP, percentage point. (DOCX) [file pmed.1002458.s007.docx]

**S5 Table. Sensitivity analysis: Proportion of participants in a peer educator group reporting each outcome. PP, percentage point.**

**Abbreviations:** CI = confidence interval; sig = significance; p-val = p-value.

*Pre-specified primary outcomes: any HIV testing at one month and at four months.

^1^Multilevel mixed effects generalized linear models, study arm fixed effect; intention-to-treat analyses.

^2^All testing and linkage to care outcomes self-reported since study start.

^3^Facility-based HIV testing included private and public healthcare facilities.

^4^For these outcomes, participants had to report both testing HIV positive and seeking HIV-related medical care or initiating ART. These outcomes were measured among all participants randomized, as defined by the intention-to-treat analysis.

| **Outcome*^2^*** | |  | ***Direct provision vs.***  ***Standard-of-care*** | | ***Facility collection vs.***  ***Standard-of-care*** | | ***Direct provision vs.***  ***Facility collection*** | | **Joint sig. test** |
| --- | --- | --- | --- | --- | --- | --- | --- | --- | --- |
|  |  | **Assessment** | **PP^1^ (95% CI)** | ***p*-val** | **PP^1^ (95% CI)** | ***p-*val** | **PP^1^ (95% CI)** | ***p*-val** | ***p*-val** |
| ***HIV testing*** | |  |  |  |  |  |  |  |  |
| Tested for HIV | | 1 month* | 24.4 (14.1 to 34.7) | <0.001 | 9.9 (-0.1 to 19.9) | 0.053 | 14.5 (4.2 to 24.8) | 0.006 | <0.001 |
|  |  | 4 months* | 13.1 (7.9 to 18.4) | <0.001 | 10.7 (5.6 to 15.7) | <0.001 | 2.5 (-2.7 to 7.8) | 0.354 | <0.001 |
|  | *Tested for HIV twice* | 4 months | 29.7 (18.9 to 40.4) | <0.001 | 15.2 (4.8 to 25.6) | 0.004 | 14.5 (3.8 to 25.2) | 0.008 | <0.001 |
| Used an HIV self-test | | 1 month | --- |  | --- |  | 15.9 (8.8 to 22.9) | <0.001 | <0.001 |
|  |  | 4 months | --- |  | --- |  | 3.8 (0.0 to 7.6) | 0.050 | <0.001 |
|  | *Used a self-test twice* | 4 months | --- |  | --- |  | 24.9 (6.4 to 43.3) | 0.008 | <0.001 |
| Tested for HIV at a facility^3^ | | 1 month | -56.6 (-66.1 to -47.0) | <0.001 | -57.1 (-66.4 to -47.9) | <0.001 | 0.6 (-8.9 to 10.0) | 0.907 | <0.001 |
|  |  | 4 months | -64.2 (-72.8 to -55.7) | <0.001 | -60.2 (-68.6 to -51.9) | <0.001 | -4.0 (-12.5 to 4.5) | 0.359 | <0.001 |
|  | *Tested for HIV at a facility twice* | 4 months | -320.9 (-389.4 to -252.3) | <0.001 | -310.3 (-376.7 to -243.9) | <0.001 | -10.6 (-78.8 to 57.5) | 0.760 | <0.001 |
| Tested HIV-positive | | 1 month | 1.2 (-6.3 to 8.8) | 0.747 | 4.6 (-2.7 to 11.9) | 0.218 | -3.3 (-10.8 to 4.1) | 0.382 | 0.445 |
|  |  | 4 months | 0.3 (-9.4 to 10.0) | 0.951 | 9.5 (0.1 to 18.9) | 0.047 | -9.2 (-18.9 to 0.4) | 0.061 | 0.081 |
| ***Linkage to care^4^*** | |  |  |  |  |  |  |  |  |
| Sought medical care for HIV | | 1 month | -2.0 (-6.8 to 2.8) | 0.411 | -3.3 (-8.0 to 1.3) | 0.154 | 1.4 (-3.4 to 6.1) | 0.575 | 0.359 |
|  |  | 4 months | -0.9 (-8.3 to 6.5) | 0.812 | 0.3 (-6.9 to 7.4) | 0.942 | -1.2 (-8.5 to 6.2) | 0.757 | 0.950 |
| Initiated ART | | 1 month | 0.5 (-3.5 to 4.4) | 0.811 | -0.4 (-4.2 to 3.4) | 0.835 | 0.9 (-3.0 to 4.8) | 0.658 | 0.906 |
|  |  | 4 months | 0.8 (-6.0 to 7.6) | 0.817 | 1.6 (-4.9 to 8.2) | 0.629 | -0.8 (-7.5 to 5.9) | 0.812 | 0.890 |
