# Supplementary material for: Direct provision versus facility collection of HIV self-tests among female sex workers in Uganda: A cluster-randomized controlled health systems trial
Source: PLoS Med. 2017 Nov 28;14(11):e1002458. doi: 10.1371/journal.pmed.1002458 (PMC5705079; doi:10.1371/journal.pmed.1002458)
Supplement: S6 Table — (DOCX) [file pmed.1002458.s008.docx]

| **Outcome^1^** | **4 months** | | |
| --- | --- | --- | --- |
|  | ***Direct provision*** | ***Facility collection*** | ***Standard-of-care*** |
| ***HIV testing*** |  |  |  |
| Tested for HIV (past month) | 156/262 (59.5%) | 185/297 (62.3%) | 139/302 (46.0%) |
| Tested for HIV (past 3 months)^2^ | 242/262 (93.4%) | 260/297 (87.6%) | 221/302 (73.2%) |
| Used an HIV self-test | 246/262 (93.9%) | 252/297 (84.9%) | 5/302 (1.7%) |
| Tested for HIV at a facility^3^ | 35/262 (13.4%) | 59/297 (19.9%) | 227/302 (75.2%) |
| Tested HIV-positive | 34/259 (13.1%) | 66/288 (22.9%) | 45/294 (15.3%) |
| ***Linkage to care^4^*** |  |  |  |
| Sought medical care for HIV | 24/260 (9.2%) | 32/289 (11.1%) | 33/294 (11.2%) |
| Initiated ART | 16/260 (6.2%) | 20/289 (6.9%) | 21/294 (7.1%) |

**S6 Table. Noncumulative outcomes at 4 months.**

^1^All testing and linkage to care outcomes self-reported since the 1-month assessment.

^2^Included testing in the past 3 months because there was a delay to the start of the 4 month assessment so that it began more than a month after we distributed the second self-tests and coupons.

^3^Facility-based HIV testing included private and public healthcare facilities.

^4^For these outcomes, participants had to report both testing HIV positive and seeking HIV-related medical care or initiating ART.
