# Supplementary material for: Direct provision versus facility collection of HIV self-tests among female sex workers in Uganda: A cluster-randomized controlled health systems trial
Source: PLoS Med. 2017 Nov 28;14(11):e1002458. doi: 10.1371/journal.pmed.1002458 (PMC5705079; doi:10.1371/journal.pmed.1002458)
Supplement: S7 Table — RR, risk ratio. (DOCX) [file pmed.1002458.s009.docx]

| **Outcome*^2^*** |  | ***Direct provision vs.***  ***Standard-of-care*** | | ***Facility collection vs.***  ***Standard-of-care*** | | ***Direct provision vs.***  ***Facility collection*** | | **Joint significance test** |
| --- | --- | --- | --- | --- | --- | --- | --- | --- |
|  | **Assessment** | **RR^1^ (95% CI)** | ***p*-value** | **RR^1^ (95% CI)** | ***p*-value** | **RR^1^ (95% CI)** | ***p*-value** | ***p-*value** |
| ***HIV testing*** |  |  |  |  |  |  |  |  |
| Tested for HIV  (past month) | 4 months | 1.29 (1.00-1.66) | 0.046 | 1.35 (1.08-1.69) | 0.009 | 0.96 (0.75-1.21) | 0.718 | 0.025 |
| Tested for HIV  (past 3 months)^2^ | 4 months | 1.26 (1.14-1.40) | <0.001 | 1.20 (1.07-1.34) | 0.002 | 1.06 (0.97-1.14) | 0.187 | <0.001 |
| Used an HIV self-test | 4 months | --- |  | --- |  | 1.11 (1.03-1.19) | 0.004 | <0.001 |
| Tested for HIV at a facility^3^ | 4 months | 0.18 (0.12-0.26) | <0.001 | 0.26 (0.20-0.35) | <0.001 | 0.67 (0.43-1.06) | 0.089 | <0.001 |
| Tested HIV-positive | 4 months | 0.87 (0.55-1.38) | 0.552 | 1.50 (0.95-2.36) | 0.080 | 0.58 (0.38-0.89) | 0.012 | 0.033 |
| ***Linkage to care^4^*** |  |  |  |  |  |  |  |  |
| Sought medical care for HIV | 4 months | 0.82 (0.49-1.39) | 0.467 | 0.98 (0.60-1.62) | 0.951 | 0.84 (0.48-1.44) | 0.519 | 0.737 |
| Initiated ART | 4 months | 0.87 (0.43-1.77) | 0.705 | 0.97 (0.50-1.88) | 0.928 | 0.90 (0.46-1.78) | 0.761 | 0.925 |

**S7 Table. Sensitivity analysis: Noncumulative outcomes at 4 months. RR, risk ratio.**

^1^Multilevel mixed effects generalized linear models (Poisson distribution, log link, robust standard errors), study arm fixed effect, peer educator random effect; intention-to-treat analyses.

^2^All testing and linkage to care outcomes self-reported since the 1-month assessment.

^3^Facility-based HIV testing included private and public healthcare facilities.

^4^For these outcomes, participants had to report both testing HIV positive and seeking HIV-related medical care or initiating ART. These outcomes were measured among all participants randomized, as defined by an intention-to-treat analysis.
