# Supplementary material for: Direct provision versus facility collection of HIV self-tests among female sex workers in Uganda: A cluster-randomized controlled health systems trial
Source: PLoS Med. 2017 Nov 28;14(11):e1002458. doi: 10.1371/journal.pmed.1002458 (PMC5705079; doi:10.1371/journal.pmed.1002458)
Supplement: S8 Table — RR, risk ratio. (DOCX) [file pmed.1002458.s010.docx]

**S8 Table. Sensitivity analysis: linkage to care among participants who reported testing HIV positive. RR, risk ratio.**

| **Outcome*^2^*** |  | ***Direct provision vs.***  ***Standard-of-care*** | | ***Facility collection vs.***  ***Standard-of-care*** | | ***Direct provision vs.***  ***Facility collection*** | |
| --- | --- | --- | --- | --- | --- | --- | --- |
|  | **Assessment** | **RR^1^ (95% CI)** | ***p*-value** | **RR^1^ (95% CI)** | ***p*-value** | **RR^1^ (95% CI)** | ***p*-value** |
| ***Linkage to care^3^, unadjusted*** | |  |  |  |  |  |  |
| Sought medical care for HIV | 1 month | 0.68 (0.39-1.20) | 0.182 | 0.38 (0.21-0.67) | 0.001 | 1.81 (0.87-3.75) | 0.111 |
|  | 4 months | 0.88 (0.62-1.24) | 0.461 | 0.66 (0.47-0.94) | 0.021 | 1.33 (0.88-2.01) | 0.182 |
| Initiated ART | 1 month | 1.00 (0.49-2.04) | 1.00 | 0.56 (0.25-1.23) | 0.146 | 1.80 (0.77-4.20) | 0.173 |
|  | 4 months | 0.95 (0.57-1.59) | 0.856 | 0.75 (0.46-1.22) | 0.240 | 1.28 (0.76-2.15) | 0.352 |
| ***Linkage to care^3^, adjusted ^4^*** | |  |  |  |  |  |  |
| Sought medical care for HIV | 1 month | 0.72 (0.45-1.17) | 0.186 | 0.41 (0.23-0.75) | 0.004 | 1.75 (0.87-3.52) | 0.114 |
|  | 4 months | 0.86 (0.63-1.18) | 0.365 | 0.66 (0.47-0.94) | 0.020 | 1.30 (0.86-1.97) | 0.208 |
| Initiated ART | 1 month | 1.10 (0.56-2.16) | 0.782 | 0.61 (0.26-1.44) | 0.260 | 1.81 (0.78-4.17) | 0.165 |
|  | 4 months | 0.97 (0.59-1.57) | 0.886 | 0.77 (0.49-1.22) | 0.270 | 1.25 (0.75-2.09) | 0.390 |

^1^Multilevel mixed effects generalized linear models (modified Poisson distribution), study arm fixed effect, peer educator random effect, robust standard errors; intention-to-treat analyses.

^2^All testing and linkage to care outcomes self-reported since study start.

^3^For these outcomes, participants had to report both testing HIV positive and seeking HIV-related medical care or initiating ART.

^4^Analysis adjusted for age, age^2^, highest level of education and monthly income.
