# Supplementary material for: Direct provision versus facility collection of HIV self-tests among female sex workers in Uganda: A cluster-randomized controlled health systems trial
Source: PLoS Med. 2017 Nov 28;14(11):e1002458. doi: 10.1371/journal.pmed.1002458 (PMC5705079; doi:10.1371/journal.pmed.1002458)
Supplement: S9 Table — (DOCX) [file pmed.1002458.s011.docx]

| **Characteristic** | ***Direct provision***  **(*n* = 44)** | ***Facility collection***  **(*n* = 80)** | ***Standard-of-care***  **(*n* = 57)** | **Total**  **(*n* = 177)** |
| --- | --- | --- | --- | --- |
| Age (median, IQR) | 31 (26-35) | 30 (27-35) | 28 (25-35) | 30 (26-35) |
| Have primary partner | 20 (45.5%) | 46 (57.5%) | 28 (52.8%) | 94 (53.1%) |
| Can read and write | 37 (84.1%) | 62 (78.5%) | 44 (83.0%) | 143 (81.3%) |
| Education  *No formal*  *Primary/Junior*  *Secondary*  *Vocational*  *Tertiary* | 4 (9.1%)  25 (56.8%)  13 (29.6%)  0  2 (4.6%) | 13 (16.3%)  43 (53.8%)  23 (28.8%)  1 (1.3%)  0 | 7 (13.2%)  25 (47.2%)  21 (39.6%)  0  0 | 24 (13.6%)  93 (52.5%)  57 (32.2%)  1 (0.6%)  2 (1.1%) |
| Own mobile phone | 46 (86.8%) | 70 (87.5%) | 43 (97.7%) | 159 (89.8%) |
| Monthly income, PPP-adjusted USD^1^  *No income*  *<$105*  *$105 to $218*  *$218 to $436*  *$436 to $873*  *>$873* | 12 (27.3%)  16 (36.4%)  13 (29.6%)  3 (6.8%)  0 | 24 (30.0%)  32 (40.0%)  19 (23.8%)  3 (3.8%)  2 (2.5%) | 10 (18.8%)  19 (35.9%)  20 (37.7%)  4 (7.6%)  0 | 46 (26.0%)  67 (37.9%)  52 (29.4%)  10 (5.7%)  2 (1.1%) |
| Years in sex work (med, IQR) | 5 (2 to 10) | 6 (4 to 10) | 4 (3 to 8) | 6 (3 to 9) |
| Client per night (med, IQR) | 5 (4 to 6) | 5 (4 to 8) | 5 (5 to 8) | 5 (4 to 8) |
| Inconsistent condom use, with clients | 15 (34.1%) | 41 (51.3%) | 14 (28.0%) | 70 (40.2%) |
| Timing of last HIV test  *>3-6 months*  *>6-12 months*  *>12-24 months*  *>24 months*  *Never tested* | 12 (27.3%)  14 (31.8%)  8 (18.2%)  5 (11.4%)  5 (11.4%) | 14 (17.7%)  19 (24.1%)  23 (29.1%)  19 (24.1%)  4 (5.1%) | 12 (22.6%)  19 (35.9%)  10 (18.9%)  9 (17.0%)  3 (5.7%) | 38 (21.6%)  52 (29.6%)  41 (23.3%)  33 (18.8%)  12 (6.8%) |
| Last HIV test facility-based^1^ | 35 (79.6%) | 57 (71.3%) | 38 (71.7%) | 130 (73.5%) |
| Intimate partner violence, past 3 months  *Physical*  *Sexual*  *Any* | 10 (22.7%)  13 (29.5%)  17 (38.6%) | 30 (37.5%)  18 (22.5%)  32 (40.0%) | 23 (44.2%)  17 (32.7%)  26 (50.0%) | 63 (35.8%)  48 (27.3%)  75 (42.6%) |

**S9 Table. Baseline descriptive characteristics for participants who reported testing HIV positive at 4 months.**

**Abbreviations:** n, total number of participants; IQR = interquartile range; med = median; PPP = purchasing power parity.

^1^Price categories in PPP-adjusted US dollars (USD); World Bank: 1 PPP-adjusted USD = 1,146 Ugandan Shillings.

^2^Includes public sector, private sector, or antenatal care clinic, other testing locations included: home, work, other.
