# Supplementary material for: Direct provision versus facility collection of HIV self-tests among female sex workers in Uganda: A cluster-randomized controlled health systems trial
Source: PLoS Med. 2017 Nov 28;14(11):e1002458. doi: 10.1371/journal.pmed.1002458 (PMC5705079; doi:10.1371/journal.pmed.1002458)
Supplement: S10 Table — (DOCX) [file pmed.1002458.s012.docx]

| **Outcome^1^** | | **1 month** | | | **4 months** | | |
| --- | --- | --- | --- | --- | --- | --- | --- |
|  |  | ***Direct provision*** | ***Facility collection*** | ***Standard-of-care*** | ***Direct provision*** | ***Facility collection*** | ***Standard-of-care*** |
| Tested for HIV at a private facility | | 10/289 (3.5%) | 12/321 (3.7%) | 128/316 (40.5%) | 19/262 (7.3%) | 35/297 (11.8%) | 171/302 (56.6%) |
|  | *Tested for HIV at a private facility twice* | --- | --- | --- | 0/262 (0%) | 2/297 (0.7%) | 69/302 (22.9%) |
| Tested for HIV at a public facility | | 17/289 (5.9%) | 16/321 (5.0%) | 83/316 (26.3%) | 37/262 (14.1%) | 44/297 (14.8%) | 135/302 (44.7%) |
|  | *Tested for HIV at a public facility twice* | --- | --- | --- | 4/262 (1.5%) | 3/297 (1.0%) | 27/302 (8.9%) |

**S10 Table. Private and public healthcare facility-based testing at 1 month and at 4 months.**

^1^All testing and linkage to care outcomes self-reported since study start.
