# Supplementary material for: Direct provision versus facility collection of HIV self-tests among female sex workers in Uganda: A cluster-randomized controlled health systems trial
Source: PLoS Med. 2017 Nov 28;14(11):e1002458. doi: 10.1371/journal.pmed.1002458 (PMC5705079; doi:10.1371/journal.pmed.1002458)
Supplement: S11 Table — RR, risk ratio. (DOCX) [file pmed.1002458.s013.docx]

| **Outcome*^2^*** | |  | ***Direct provision vs.***  ***Standard-of-care*** | | ***Facility collection vs.***  ***Standard-of-care*** | | ***Direct provision vs.***  ***Facility collection*** | | **Joint significance test** |
| --- | --- | --- | --- | --- | --- | --- | --- | --- | --- |
|  |  | **Assessment** | **RR^1^ (95% CI)** | ***p*-value** | **RR^1^ (95% CI)** | ***p*-value** | **RR^1^ (95% CI)** | ***p*-value** | ***p-*value** |
| Tested for HIV at a private facility | | 1 month | 0.08 (0.04-0.18) | <0.001 | 0.09 (0.05-0.18) | <0.001 | 0.91 (0.36-2.31) | 0.843 | <0.001 |
|  |  | 4 months | 0.13 (0.07-0.23) | <0.001 | 0.21 (0.14-0.31) | <0.001 | 0.61 (0.31-1.21) | 0.158 | <0.001 |
|  | *Tested for HIV at a private facility twice* | 4 months | 0.00 (0.00-0.00) | <0.001 | 0.03 (0.01-0.12) | <0.001 | 0.00 (0.00-0.00) | <0.001 | <0.001 |
| Tested for HIV at a public facility | | 1 month | 0.22 (0.12-0.42) | <0.001 | 0.19 (0.10-0.35) | <0.001 | 1.18 (0.55-2.50) | 0.673 | <0.001 |
|  |  | 4 months | 0.31 (0.21-0.48) | <0.001 | 0.33 (0.24-0.46) | <0.001 | 0.95 (0.61-1.49) | 0.833 | <0.001 |
|  | *Tested for HIV at a public facility twice* | 4 months | 0.18 (0.06-0.53) | 0.002 | 0.12 (0.03-0.39) | <0.001 | 1.54 (0.36-6.56) | 0.560 | <0.001 |

**S11 Table. Effect size estimates: Public and private healthcare facility-based testing. RR, risk ratio.**

^1^Multilevel mixed effects generalized linear models (Poisson distribution, log link, robust standard errors), study arm fixed effect, peer educator random effect; intention-to-treat analyses

^2^All testing and linkage to care outcomes self-reported since study start.
